# An MCM family protein promotes interhomolog recombination by preventing precocious intersister repair of meiotic DSBs

**DOI:** 10.1371/journal.pgen.1008514

**Published:** 2019-12-09

**Authors:** Miao Tian, Josef Loidl

**Affiliations:** Department of Chromosome Biology, Max F. Perutz Laboratories, University of Vienna, Vienna, Austria; University of Chicago, UNITED STATES

## Abstract

Recombinational repair of meiotic DNA double-strand breaks (DSBs) uses the homologous chromosome as a template, although the sister chromatid offers itself as a spatially more convenient substrate. In many organisms, this choice is reinforced by the recombination protein Dmc1. In *Tetrahymena*, the repair of DSBs, which are formed early in prophase, is postponed to late prophase when homologous chromosomes and sister chromatids become juxtaposed owing to tight parallel packing in the thread-shaped nucleus, and thus become equally suitable for use as repair templates. The delay in DSB repair is achieved by rejection of the invading strand by the Sgs1 helicase in early meiotic prophase. In the absence of Mcmd1, a meiosis-specific minichromosome maintenance (MCM)-like protein (and its partner Pamd1), Dmc1 is prematurely lost from chromatin and DNA synthesis (as monitored by BrdU incorporation) takes place in early prophase. In *mcmd1*Δ and *pamd1*Δ mutants, only a few crossovers are formed. In a *mcmd1*Δ *hop2*Δ double mutant, normal timing of Dmc1 loss and DNA synthesis is restored. Because *Tetrahymena* Hop2 is believed to enable homologous strand invasion, we conclude that Dmc1 loss in the absence of Mcmd1 affects only post-invasion recombination intermediates. Therefore, we propose that the Dmc1 nucleofilament becomes dismantled immediately after forming a heteroduplex with a template strand. As a consequence, repair synthesis and D-loop extension starts in early prophase intermediates and prevents strand rejection before the completion of homologous pairing. In this case, DSB repair may primarily use the sister chromatid. We conclude that Mcmd1‒Pamd1 protects the Dmc1 nucleofilament from premature dismantling, thereby suppressing precocious repair synthesis and excessive intersister strand exchange at the cost of homologous recombination.

## Author summary

Minichromosome maintenance (MCM) proteins are mainly known for their involvement in DNA replication. However, distant members of this protein family have recently been shown to promote interhomolog over intersister recombination in meiosis. They achieve this by enforcing or stabilizing the invasion of a double-stranded DNA by a filament consisting of a homologous single-stranded DNA molecule coated with a strand exchange protein. This interaction then would lead to the exchange of DNA strands and, ultimately, crossing over. Here, we study a member of the MCM protein family in the protist *Tetrahymena thermophila*. Meiosis in this organism has several unusual features: A synaptonemal complex is not formed, and homologous prealignment occurs during the close parallel arrangement of chromosomes in the extremely elongated, threadlike meiotic prophase nucleus. This noncanonical pairing has come along with altered mechanisms for recombination partner choice. Thus, we find that the *Tetrahymena* meiotic MCM protein promotes crossovers in an unprecedented way: It suppresses the formation of recombination intermediates between sister DNA molecules early in meiosis, thereby increasing the chance of competing interhomolog recombination events. Thus, members of the same protein family have been harnessed by different organisms to achieve the same result via completely different mechanisms.

## Introduction

Meiotic crossovers (COs) are initiated by programmed DNA double-strand breaks (DSBs). DNA ends flanking a DSB are resected to produce single-stranded (ss) 3´-overhangs. These ssDNA tracts are loaded with Rad51 and/or Dmc1, and the resulting nucleofilament can invade double-stranded DNA to search for a matching base sequence. Heteroduplex formation between the invading strand and the complementary strand serves as a mechanism for homology recognition and results in the formation of a three-way structure, the D-loop. The invading strand serves as the primer for DNA synthesis, which may lead to extension and stabilization of the D-loop. At this point, either the invading strand can be rejected and rejoin with the other end, leading to a noncrossover (NCO), or the second resected end may be captured by the D-loop, and the resulting double Holliday junction may be further processed into a CO. In both cases, DNA repair synthesis and ligation fill and seal the gap left from strand resection (see e.g. [1–5]). After strand exchange, Rad51/Dmc1 remains associated with the heteroduplex, but for DNA synthesis to start at the OH-end of the invading strand these proteins must be stripped from the heteroduplex [6,7].

**Fig 1:**
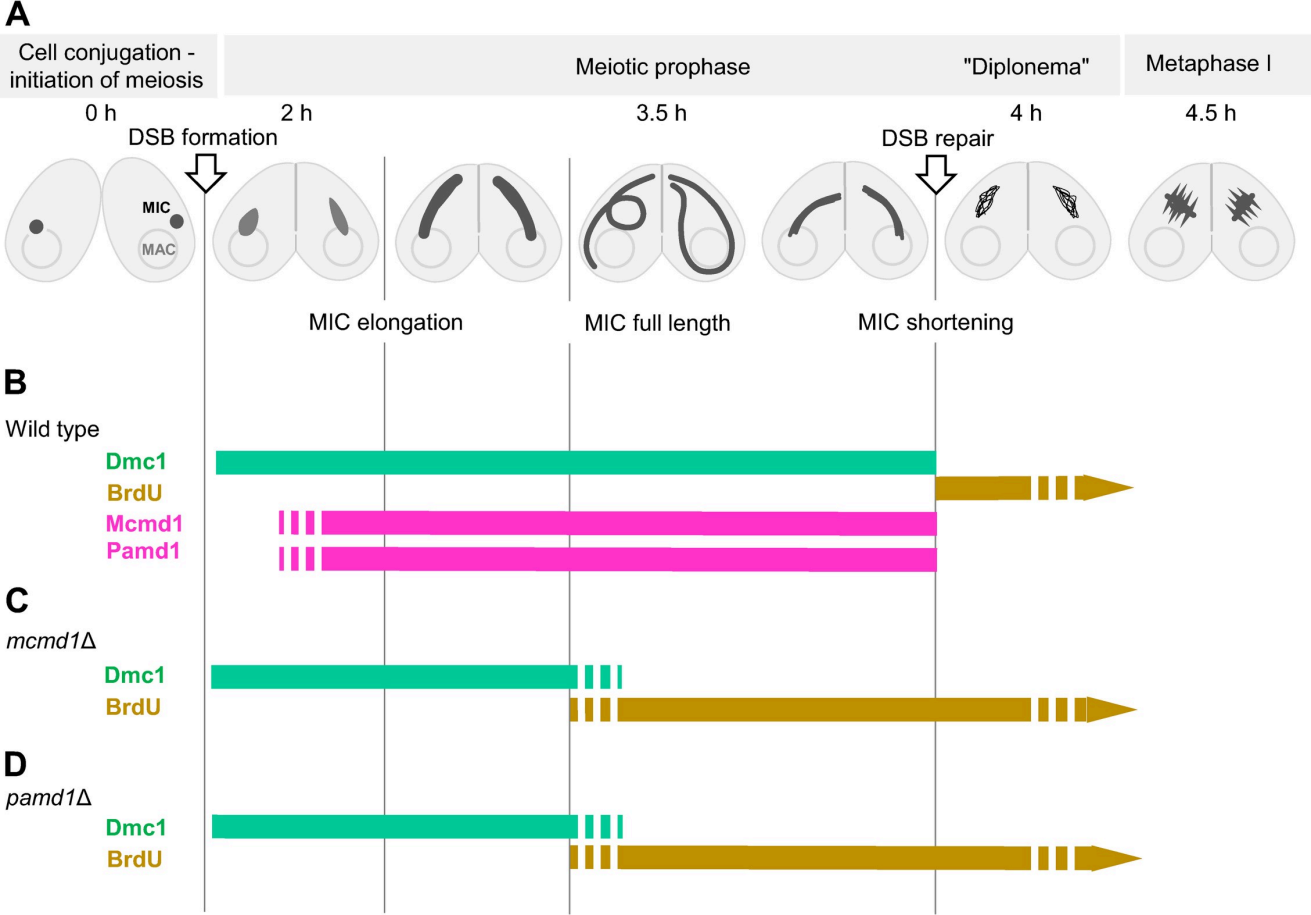
Timing of meiotic events in the wild type and in mutants. (A) Cell mating (conjugation) begins immediately after the mixing of starved cells of different mating types. Each cell possesses a polyploid somatic nucleus (the macronucleus–MAC) and a diploid germline nucleus (the micronucleus–MIC), the latter of which undergoes meiosis. Programmed meiotic DSBs occur within 2 h after mixing and trigger elongation of the MIC [15]. Meioses in the two conjugating cells progress fairly synchronously. About 3.5 h after mixing, the MIC is fully elongated to about twice the length of the cell. During elongation, the pairing of homologous loci increases [15]. After that, the MIC gradually contracts and enters a stage resembling the diplonema of canonical meiosis, which is characterized by the formation of distinct chromatin threads. About 4.5 h after mixing, five bivalents appear in the wild type, arrange in a metaphase I plate, and are separated in a closed first meiotic division. (B) In the wild type, Dmc1 appears soon after MIC elongation begins and disappears at the onset of diplonema. At this time point, BrdU is incorporated, indicating recombinational repair synthesis [13]. Mcmd1 and Pamd1 first appear in the elongating MIC and disappear at the onset of diplonema. (C, D) In *mcmd1*Δ (C) and *pamd1*Δ (D) cells, Dmc1 appears normally but has completely disappeared by the time the micronucleus has fully elongated. At this time point, the first foci of incorporated BrdU appear.

**Fig 2:**
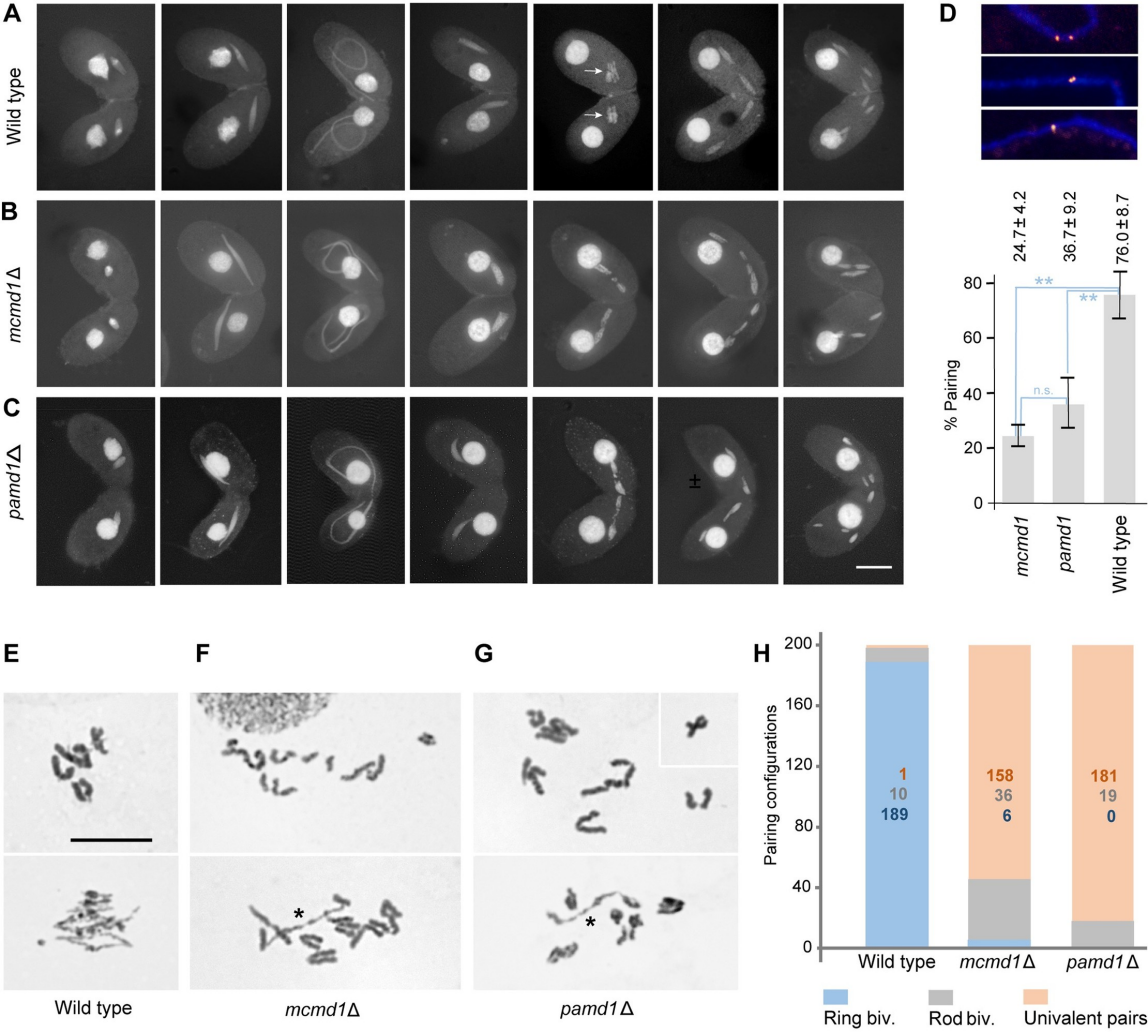
Meiotic stages and chromosome configurations in wild-type and mutant cells. (A‒C) Meiotic progression in (A) wild-type, (B) *mcmd1*Δ, and (C) *pamd1*Δ cells. Arrows in (A): Metaphase plate. (D) Pairing of homologous loci marked by FISH, with examples of single signals and two joined signals (scored as paired), and two separate signals (scored as unpaired). Error bars represent the SD from three counts of 50 nuclei each. Paring was significantly reduced in *mcmd1*Δ (t-test: p-value = 0.003248) and *pamd1*Δ (t-test: p-value = 0.005888) cells compared to wild-type cells, and did not differ significantly (n.s.) between the two mutants (t-test: p-value = 0.1398). (E‒H) Diakinesis‒metaphase I configurations (Giemsa staining). (E) Diakinesis (top) and metaphase (bottom) in the wild type with five bivalents. At diakinesis centromeres are stretched out with thin tips due to the start of microtubule attachment. Bivalent arms on both sides of the centromeres are in close contact, suggesting the presence of chiasmata in both arms. During metaphase, bivalents are arranged in a metaphase plate. Most of them are ring-shaped, indicating chiasma formation in both arms. (F) Examples of the *mcmd1*Δ diakinesis‒metaphase stages. Chromosomes mainly form univalents. (G) Examples of the *pamd1*Δ diakinesis‒metaphase stages with univalents. Univalents never assemble into a metaphase plate and become prematurely oriented toward the poles in a random fashion. The asterisks denote rod bivalents. Bars: 10 μm. (H) Quantification of bivalent (biv.) and univalent configurations. A total of 200 chromosome pairs were evaluated for each genotype.

The unicellular protist *Tetrahymena thermophila* is a convenient model organism for studying DSB repair because it has a simplified meiosis with a slimmed CO pathway and does not feature a synaptonemal complex (see [8]). *Tetrahymena* possesses a polyploid somatic macronucleus and a diploid (2n = 10) germline micronucleus within a single cell. Only the germline nucleus undergoes meiosis, and this is induced by mixing starved cells of complementary mating types. Pairs of mating *Tetrahymena* cells undergo synchronous meioses [9], and meiotic progression can be easily followed cytologically and staged (Figs 1A and 2A). The early steps in meiotic recombination follow the canonical pathway, with Spo11 inducing DSBs and strand exchange requiring Rad51 and Dmc1. Notably, only Dmc1 is visible as cytological foci, suggesting that little if any Rad51 is involved in nucleofilament formation with DNA [10,11]. COs are formed via a single Mus81-dependent pathway, which in most other model systems constitutes a secondary route to COs [12]. A notable feature of *Tetrahymena* meiosis is the enormous elongation of the meiotic prophase nucleus. Nuclear elongation begins ~2 h after meiosis induction. Within an elongated nucleus, chromosomes are arranged in a stretched bouquet-like manner, with centromeres and telomeres attached to opposite ends. This ultimate bouquet arrangement is believed to promote the juxtaposition of homologous regions and, thereby, homologous pairing and crossing over [13,14]. Following this unusual pairing stage, nuclei shorten and DSBs are repaired [13]. Condensed bivalents become discernible at the diakinesis stage, which is followed by closed first and second meiotic divisions.

Nuclear shape is a good proxy for the substages of meiotic prophase (Fig 1A and [8]): DSBs (which can be detected by the formation of Dmc1 and γ-H2A.X foci under microscopy or as electrophoretic fragments—[11]) are formed during early prophase when the nucleus starts to elongate. Full elongation to about twice the length of the cell represents mid prophase. DSB repair (as monitored by the disappearance of Dmc1 and γ-H2A.X) occurs only when the nucleus has shortened to about half the cell length and chromatin begins to condense. Repair synthesis can be detected by incorporation of the thymidine analog BrdU [13] at this stage, which may be comparable to early diplonema. Thus there is a ~2 h interval between DSB formation and repair, during which chromosome homology is believed to be probed in repeated strand invasion and rejection cycles [12].

Minichromosome maintenance (MCM) proteins are conserved in eukaryotes and form heteromultimers that function as helicases during replication (see [16]), but specific members of this protein family function in recombinational DNA repair and in meiosis (see [17]). In addition, divergent MCM family members have meiosis-specific functions (mei-MCMs—[18]). Here we report that a meiosis-specific MCM protein and its partner control the onset of recombination-related DNA synthesis in *Tetrahymena*.

## Results

### *mcmd1*Δ and *pamd1*Δ cells undergo abnormal meiosis

*MCMD1* (MCM Domain1, TTHERM_01207610 in the Tetrahymena Genome Database; http://ciliate.org; NCBI Gene ID: 7836088) is transcribed exclusively during meiotic prophase ([19]; http://tfgd.ihb.ac.cn/) and is predicted to encode a 780 aa MCM2/3/5 family protein (NCBI XP_001021907.2). A knockout mutant lacking the *MCMD1* gene was produced as part of a systematic mutant screen for genes with a function in meiosis. DAPI staining over a meiotic time course showed that nuclear elongation was normal in the mutant. However, after nuclear shortening, condensed chromosomes did not arrange in a metaphase plate (Fig 2B). Since this failure could be due to a pairing defect, we studied pairing of a homologous locus by fluorescence in situ hybridization (FISH) and indeed found reduced association of homologous loci in elongated nuclei (Fig 2D). Inspection of Giemsa-stained chromosome pairing configurations at diakinesis/metaphase I revealed that mainly univalents were formed (Fig 2F and 2H), and that these immediately oriented towards the poles at random without assembling in a metaphase plate. As a consequence, chromosome segregation at meiosis I was irregular and daughter nuclei received incorrect chromosome sets. These nuclei underwent a second meiotic division (Fig 2B), but chromosomally balanced gametic nuclei were rarely formed. Therefore, after 24 h, 93 out of 100 cells ceased the sexual reproduction process.

Pamd1 (Partner of Mcmd1) was identified by mass spectrometry as the most reliable co-IP partner of Mcmd1 (S1 Fig and S1 Table). Pamd1 is encoded by ORF TTHERM_001295283 (transcript ID: gene_000012891 - http://ciliate.org and http://tfgd.ihb.ac.cn/). Like Mcmd1, Pamd1 is expressed only in meiosis (http://tfgd.ihb.ac.cn/). Reciprocal co-IP with tagged Pamd1 as the bait produced Mcmd1 as the first significant hit in mass spectrometry (S1 Fig and S1 Table). *PAMD1* was knocked out, and *pamd1*Δ meiosis showed the same pairing and segregation irregularities and defective bivalent formation as *mcmd1*Δ (Fig 2C, 2D, 2G and 2H).

### Structural features of Mcmd1 and its partner Pamd1

**Fig 3:**
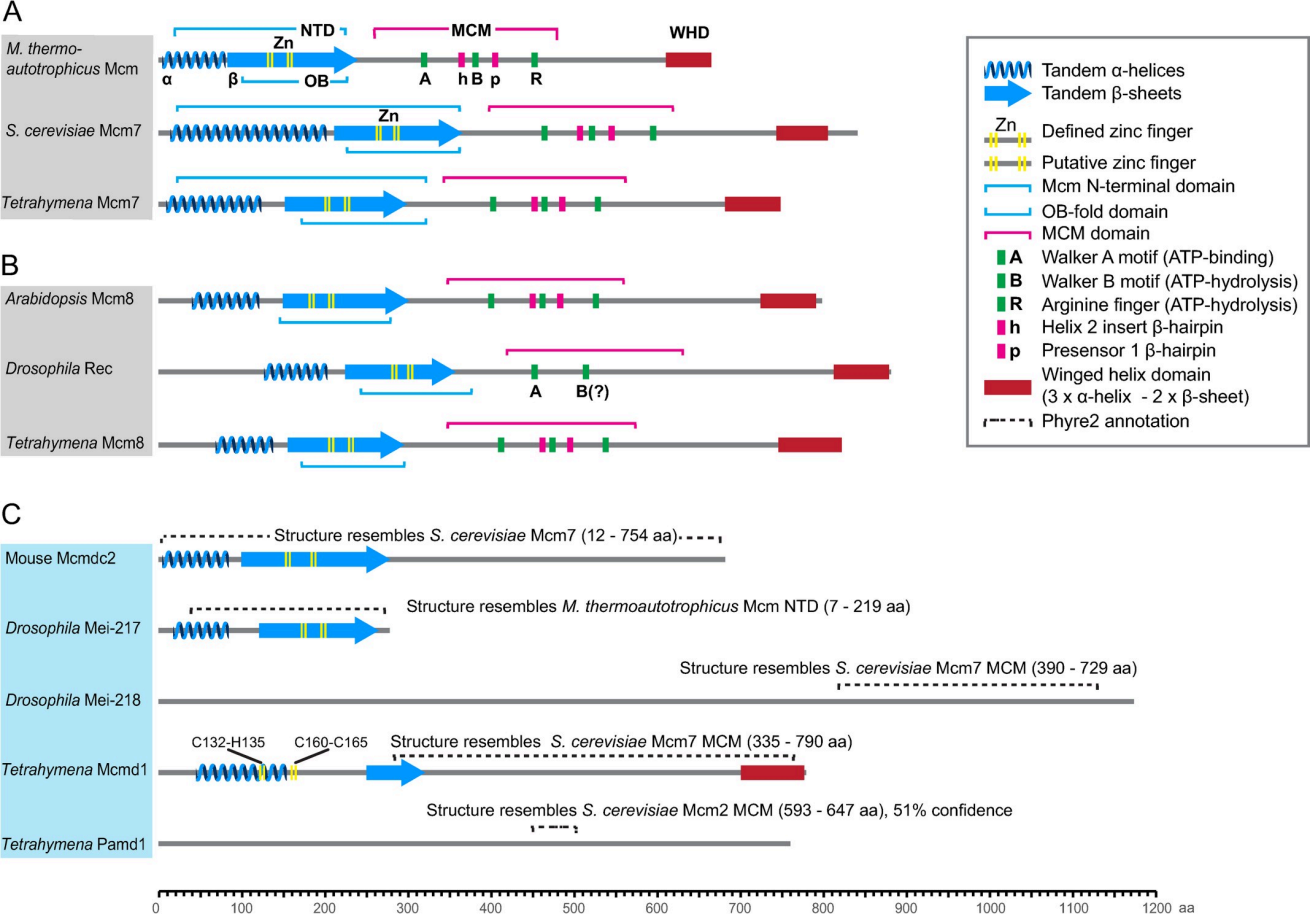
Domain organization of MCM-family proteins. (A) Representative members of core MCM family proteins including the Archaean MCM (here: *Methanothermobacter thermautotrophicus*) and eukaryotic Mcm2-7 replicative helicases, represented by budding yeast (*Saccharomyces cerevisiae*) and *Tetrahymena* Mcm7. The proteins have a conserved structure consisting of a conserved N-terminal domain (NTD), an MCM domain (also called AAA+ ATPase domain), and a C-terminal winged helix domain (WHD). The NTD consists of tandem α-helices, a zinc finger, and an oligonucleotide/oligosaccharide-binding fold (OB-fold) consisting of tandem β-sheets. The MCM domain contains conserved Walker A, Walker B, and Arginine finger motifs that are required for ATPase activity. It also has a Helix 2 insert β-hairpin and a presensor 1 β-hairpin that are required for DNA unwinding and DNA binding, respectively. (B) Examples of Mcm8 proteins. Mcm8 sequences are divergent from those of MCM replicative helicases, but their secondary structures are similar. Mcm8 proteins have a conserved MCM domain (except for *Drosophila* Rec) and WHD. (C) Examples of meiotic MCM domain proteins. The meiotic MCM domain is structurally similar to the core MCM domain (Phyre2 prediction, 100% confidence) but lacks the signature motifs that are required for ATPase activity, DNA unwinding, and DNA binding. *Drosophila* Mei-217 and Mei-218 probably originated from the splitting of an ancestral MCM protein [18], with Mei-217 carrying the NTD and Mei-218 the MCM domain. *Tetrahymena* Mcmd1 shares a somewhat greater primary sequence homology with MCM replicative helicases compared with the others, whereas Pamd1 is the most divergent protein. Pamd1 has a short region with weak structural similarity (Phyre2 prediction, 51% confidence) to the MCM domain, making an evolutionary common origin doubtful. OB-folds were annotated using the Gene3D prediction tool of the InterProScan software package [21]. MCM domains were annotated using Pfam prediction (InterProScan). α-helices and β-sheets were annotated using the JURY results from the Jpred server [22]. Structural similarity analysis was done with Phyre2 [23].

Mcmd1 is homologous to MCM proteins in animals and in budding yeast (S2 Table) and its domain organization shows that it is derived from canonical MCM proteins (Fig 3). The protein contains tandem α-helices, a putative zinc finger, and tandem β-sheets at the N-terminal region. However, this putative OB-fold does not feature charged amino-acids at the hinges, which are characteristic DNA-binding motifs of core MCM family members. The N-terminus also has a cluster of five S/T-Q motifs that are potential phosphotargets of ATM/ATR kinases [20]. The central region is structurally similar to the MCM (AAA+ ATPase) domain (Phyre2 prediction, 100% confidence), but does probably not function as ATPase. The C-terminus has three tandemly arranged α-helices followed by two β-sheets, which resembles the winged helix domain (WHD) of the core MCM proteins.

Pamd1 has only a small region that shares weak structural similarity with the MCM domain (Fig 3C). Apart from this, it does not possess any conserved domain or sequence homology to non-*Tetrahymena* proteins. Therefore, its evolutionary descent from a core MCM protein is doubtful.

### Mcmd1 and Pamd1 localize to meiotic prophase nuclei

**Fig 4:**
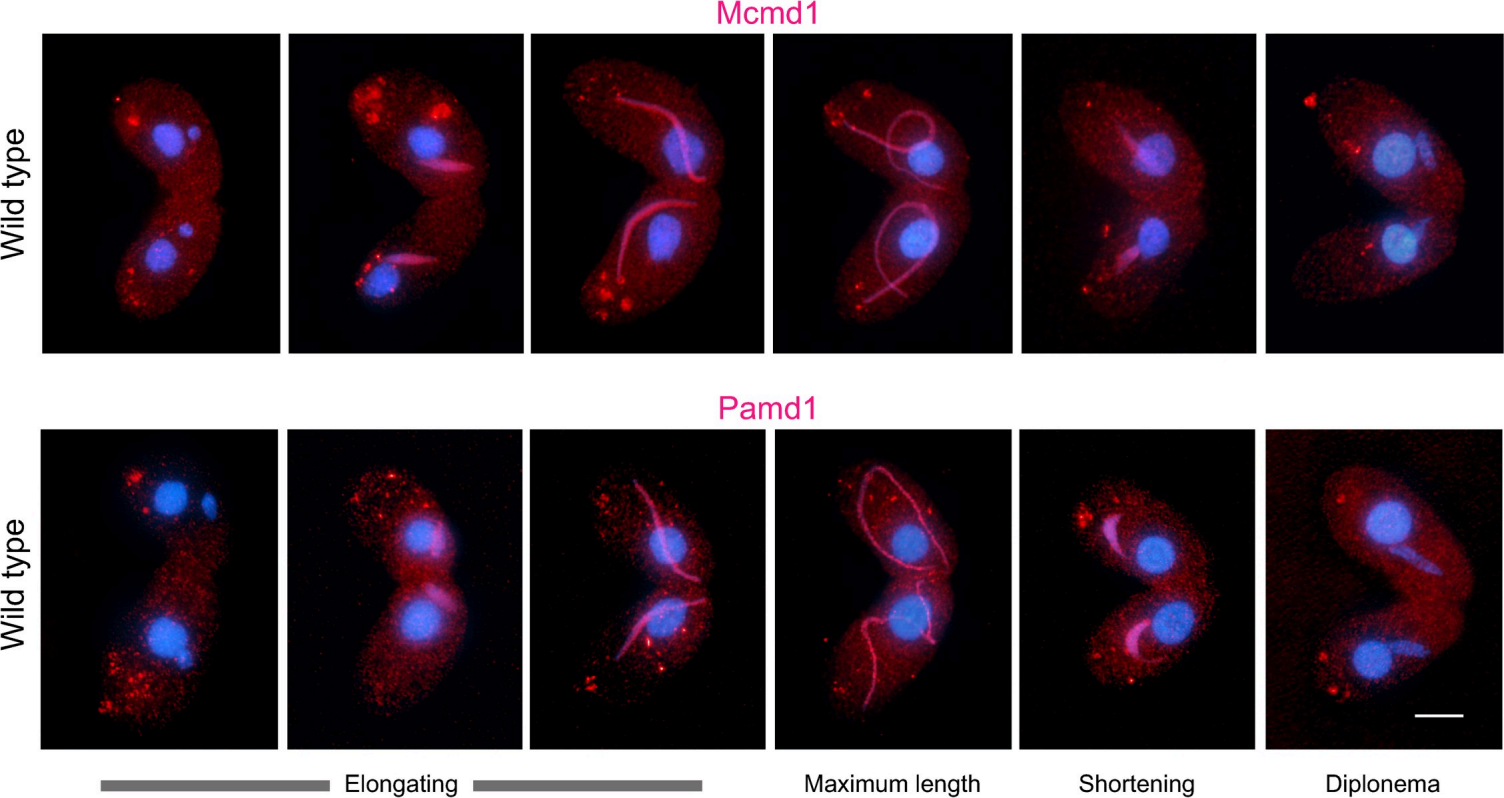
Wild-type localization of Mcmd1 and Pamd1. Mcmd1-HA and Pamd1-HA appear shortly after nuclei begin to elongate, are most abundant in fully elongated nuclei, disappear from shortening nuclei, and are completely gone by diplonema. This timing is the same as for Dmc1 foci (Fig 5), but Mcmd1 and Pamd1 show uniform nuclear distribution rather than foci. Bar: 10 μm.

To detect the localization of Mcmd1 and Pamd1, we labeled the two proteins with a C-terminal hemagglutinin (HA) tag. Both proteins localized only to the germline nucleus during meiotic prophase (Figs 1B and 4). Unlike Dmc1 (representing nucleofilaments at DSBs) and BrdU (representing newly synthesized DNA at DSBs) Mcmd1 and Pamd1 do not form distinct foci, suggesting that their localization to chromatin is not limited to DSB sites. We created *PAMD1-HA mcmd1*Δ and *MCMD1-HA pamd1*Δ strains and mated them to test whether nuclear localization of the two proteins is mutually dependent. From the elongating to fully elongated nuclei stages, neither protein was found in the absence of the other (100 nuclei evaluated for each genotype). For controls, *PAMD1-HA mcmd1*Δ × wild type and *MCMD1-HA pamd1*Δ × wild-type matings were performed. Since mating cells can exchange proteins [24], both cells in a mating pair were phenotypically Pamd1-HA Mcmd1^+^ and Mcmd1-HA Pamd1^+^, respectively. We found that half the wild-type protein dosage of Mcmd1 or Pamd1 was sufficient to support localization of the partner protein in both mating cells. Mcmd1 was found in 98% of fully elongated nuclei, whereas Pamd1 was found in 77% (100 nuclei were counted for each phenotype). The large proportion of nuclei without Pamd1 staining does not indicate that Pamd1 localizes only at a specific part of the full elongation stage because preceding and following stages showed Pamd1 staining (Fig 4); instead, the absence of staining may be due to low abundance of the protein. Neither of the proteins were visible in diplotene nuclei. In conclusion, localization of both proteins is mutually dependent, and we conclude that they function in the same pathway, most likely in a complex.

### Dmc1 removal from chromatin and repair synthesis occur prematurely in *mcmd1*Δ and *pamd1*Δ

**Fig 5:**
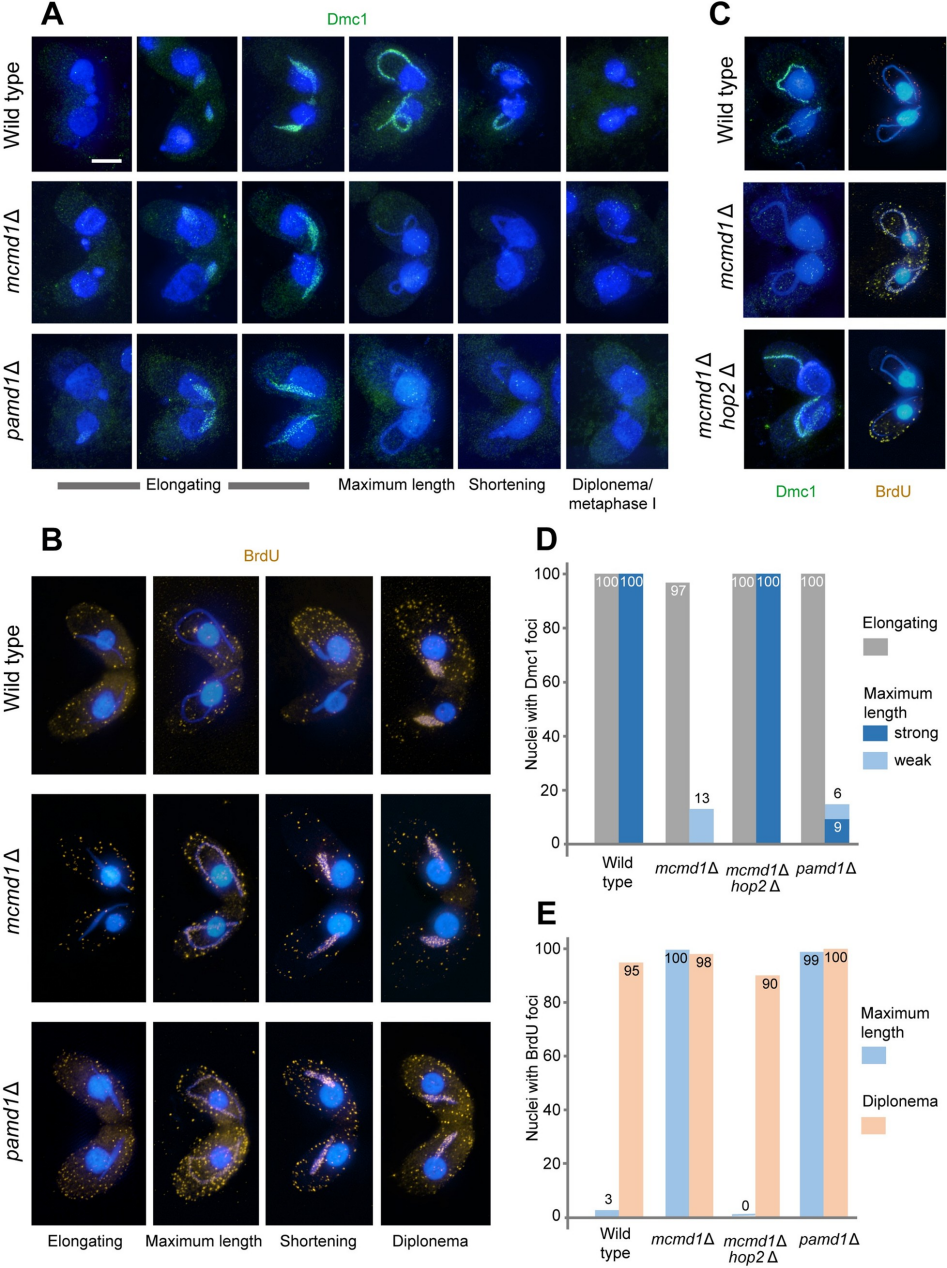
Timing of Dmc1 and BrdU. (A) Dmc1 foci (green) in wild-type, *mcmd1*Δ, and *pamd1*Δ cells. Dmc1 is present from the elongation stage throughout the shortening stage in the wild type, but only in elongating nuclei in the mutants. Cells were fixed by high-detergent treatment to remove free nuclear Dmc1, which would otherwise persist to anaphase II. (B) BrdU foci (ochre) in wild-type, *mcmd1*Δ, and *pamd1*Δ cells. BrdU is not incorporated before diplonema in the wild type but is incorporated as early as the fully elongated nucleus stage in the mutants. (C) In the *mcmd1*Δ *hop2*Δ double mutant, the wild-type dynamics of Dmc1 disappearance and BrdU incorporation is restored. (D). Staging of Dmc1 expression. (E) Staging of BrdU incorporation. Bar: 10 μm.

The recombination protein Dmc1 localizes to meiotic prophase nuclei (Figs 1B and 5A and [11]). Since the appearance of Mcmd1, Pamd1 and Dmc1 coincide, loading and localization of all three proteins may be interdependent. Therefore, we studied Dmc1 localization in the absence of Mcmd1 and Pamd1 (Fig 5). Interestingly, loading of Dmc1 is normal in the two mutants but it disappears prior to full elongation of the nucleus; in contrast, in wild-type cells Dmc1 remains on chromatin into the nuclear shortening stage (Figs 1B, 1C, 1D, 5A and 5D). (For this experiment, cells were fixed under high-detergent conditions, which removes free nuclear Dmc1 –see Materials and Methods). Since Dmc1 is lost from chromatin at the onset of recombination-related DNA synthesis (see [8]), we asked whether the precocious removal of Dmc1 in the mutants is coincident with the untimely onset of DNA synthesis. For this, we fed cells with BrdU 2.5 h after mixing (i.e. when nuclei begin to elongate) and fixed cells and immunostained incorporated BrdU at timepoints corresponding to various stages of prophase. In agreement with previous observations, BrdU was incorporated only into diplotene nuclei of the wild type (Fig 5B). However, in both mutants, BrdU incorporation (and hence DNA synthesis) began in fully elongated nuclei (Figs 1C, 1D, 5B and 5E). Thus, we conclude that loss of Dmc1 is coordinated with the onset of recombinational repair synthesis and suggest that, in the absence of Mcmd1‒Pamd1, Dmc1 is lost too early, causing the precocious onset of DNA synthesis.

A possible explanation for untimely DNA synthesis in the absence of Dmc1 is that Rad51 takes over and repairs DSBs via the sister chromatid. Sister chromatid-dependent repair seems to require only short Rad51 nucleofilaments, which are below the threshold for cytological detection [11]. Thus, failure to detect Rad51 in fully elongated nuclei does not exclude this possibility. If Rad51-dependent repair could substitute for Dmc1, then precocious BrdU incorporation would be observed also in a *dmc1*Δ mutant. However, this was not the case (S2 Fig).

Next, we wanted to know whether Mcmd1‒Pamd1 protects DNA‒Dmc1 complexes from dismantling prior to or after strand invasion. Hop2 is known to promote homologous strand invasion in other eukaryotes [25–27], and this function of Hop2 is conserved in *Tetrahymena* [15]. Therefore, we created a *mcmd1*Δ *hop2*Δ mutant in which strand invasion by ssDNA‒Dmc1 nucleofilaments is prevented. Notably, in this double mutant, precocious Dmc1 removal and DNA synthesis did not occur (Fig 5C–5E). This result suggests that Mcmd1‒Pamd1 does not protect the ssDNA‒Dmc1 complex from dismantling prior to strand invasion but instead prevents untimely Dmc1 removal from joint molecules (JMs).

Finally, we asked whether early onset of DNA repair in *mcmd1*Δ leads to the early completion of repair. γ-H2A.X (a phosphorylated histone H2A variant) is a marker for DSBs [28]. In wild-type *Tetrahymena* the γ-H2A.X signal disappears in diplonema [15,29], consistent with DSB repair being completed by this stage. To directly compare γ-H2A.X intensity in the elongated nuclei of *mcmd1*Δ and wild-type cells, side-by-side mating pairs were scored using fluorescence-tagged histone H3 (Hht2-mCherry) as a marker for wild-type cells. We found that γ-H2A.X is not notably reduced in *mcmd1*Δ fully elongated nuclei (S3 Fig). Therefore, despite the precocious onset, DSB repair is not completed earlier in *mcmd1*Δ than in the wild type. Furthermore, the relatively weak BrdU signal seen in fully elongated *mcmd1*Δ nuclei may also indicate that early-onset DNA synthesis is not complete at this stage.

## Discussion

### Mcmd1 and Pamd1 prevent untimely DSB repair synthesis

**Fig 6:**
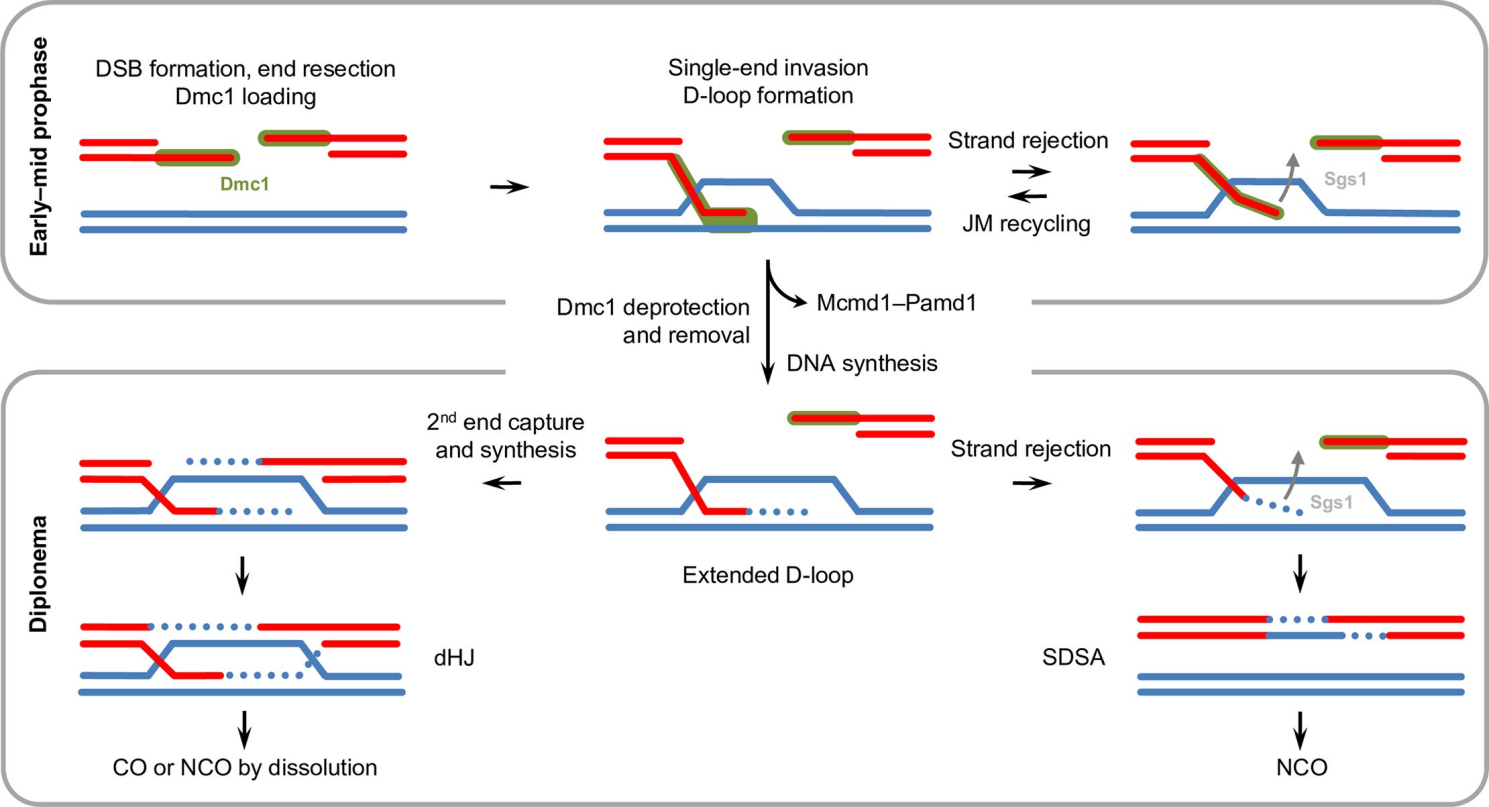
Model of D-loop progression. Single-stranded 3´ overhangs at DSB ends become loaded with Dmc1 (green). One end can invade a DNA molecule to probe for homology and form a Dmc1‒heteroduplex strand. Early joint molecules (JMs) will mainly be formed between sister DNA molecules and are unstable. They can reject the invading strand with the help of Sgs1 and then re-use it in subsequent rounds of homology testing. In diplonema, the heteroduplex is stripped of Dmc1, allowing DNA synthesis (dotted line) to begin at the OH-end of the invading strand. This will extend the D-loop. The extended strand can then be displaced, pair with the other end of the DSB, fill the gaps (via synthesis-dependent strand annealing ‒ SDSA), and produce a noncrossover (NCO). Alternatively, the extended D-loop may capture the second DSB end and form a double Holliday junction (dHJ). This intermediate may either become a NCO by dissolution or mature into a crossover (CO). In the absence of Sgs1, early JMs will persist into diplonema and be then transformed into (mainly intersister) COs. In the absence of Mcmd1‒Pamd1, early JMs will be immediately converted to extended D-loops and also result primarily in intersister exchange.

The untimely loss of Dmc1 and onset of DNA synthesis in *mcmd1*Δ and *pamd1*Δ mutants suggest that Mcmd1 and Pamd1 retard these processes in the wild type. (For a summary of the dynamic localization of Dmc1, Mcmd1, Pamd1, and BrdU, see Fig 1) In vitro and in vivo experiments in different organisms have shown that RecA/Rad51/Dmc1 must be removed from a heteroduplex after strand exchange to allow extension of the invading strand by DNA synthesis [6,7,30,31]. We propose a model in which an Mcmd1‒Pamd1 complex counteracts an activity that sheds Dmc1 from the heteroduplex (Fig 6). During early to mid-prophase, this protection of Dmc1 prevents the initiation of repair synthesis and thus the stabilization of (primarily intersister) JMs. At this stage, repeated strand invasion and rejection events in the search for DNA homology gradually involve more interhomolog events as homolog juxtapositioning increases during nuclear elongation. By diplonema, when homologs are fully aligned, a sufficiently large proportion of D-loops will be interhomologous. Dmc1 protection by Mcmd1‒Pamd1 then ceases and DNA synthesis can begin. The elongated strands can be rejected and DSB repair occurs by synthesis-dependent strand annealing (SDSA) or (in some intermediates) the second DSB end can be captured and elongated, leading to a double Holliday junction and eventually a CO (Fig 6). The reduced homologous pairing in elongated nuclei (Fig 2D) along with the formation of intact univalents at metaphase I (Fig 2F and 2G) in *mcmd1*Δ and *pamd1*Δ mutants are consistent with the precocious termination of homology searching and efficient DSB repair via the sister chromatid.

The protection of dsDNA‒Dmc1 complexes by Mcmd1‒Pamd1 raises the question of how these complexes may subsequently become dismantled to enable repair synthesis. Swi2/Snf2 family proteins with appropriate DNA translocase or strippase activities have been reported in various organisms [31]. In budding yeast, Rad54 is the primary factor in the analogous Rad51-directed activity [32]. In *C*. *elegans*, RFS-1 and HELQ-1 contribute redundantly to Rad51 removal via distinct mechanisms, possibly with help from RAD-54 [7]. However, in *Tetrahymena*, no homologs of these proteins were identified as co-IP partners of Mcmd1 or Pamd1 (S1 Table).

### Early JMs may evade D-loop extension and be resolved by Sgs1

The observations of defective homologous pairing and almost exclusive univalent formation in *mcmd1*Δ and *pamd1*Δ suggest that the timing of JM maturation is crucial for the intersister vs. interhomolog repair decision. We cannot exclude the possibility that the early-repaired intermediates in the absence of Mcmd1‒Pamd1 are channeled into the interhomolog SDSA pathway. However, as homologous associations increase only during nuclear elongation [15], it is reasonable to assume that most early repair events are intersister. This interpretation is consistent with the different fates of DSBs in budding yeast, where early DSBs that function in homology searching are repaired mostly via the sister, whereas later DSBs have an interhomolog bias [33]. Our observation that the repair of early DSBs is retarded in *Tetrahymena* supports the idea that early (primarily intersister) JMs are unwound by Sgs1 so that the single strands can be re-used in ongoing homology testing and, finally, in CO formation in diplonema, when homologs are tightly paired (Fig 6, [12]). In the absence of Sgs1, fully elongated nuclei retain Dmc1 [34] and BrdU foci are absent (S4 Fig). These findings suggest that dsDNA‒Dmc1 complexes, some between sisters, are preserved and that intersister JMs are probably transformed into intersister COs at diplonema [34]. In the absence of Mcmd1‒Pamd1 early intersister JMs could be stabilized by starting DNA synthesis before the invading strand can be rejected and processed further to NCOs or intersister COs. According to this model, strand rejection would take place at two stages of D-loop development: First, prior to strand elongation, such that it may be re-used for homology probing; and second, after the strand has copied a sequence from the template, which enables it to bridge the DSB by SDSA (Fig 6). Similar models for a function of Sgs1 in recycling early D-loops were proposed by [35], [36] and [37].

We tested a possible genetic interaction of *SGS1* and *MCMD1* by creating a *sgs1*(RNAi) *mcmd1* double mutant (S1 Text). We found that, like in the *mcmd1* mutant, Dmc1 precociously disappeared from fully elongated nuclei, repair synthesis began at that stage, and univalents were formed. Altogether, our findings indicate that both Sgs1 and Mcmd1‒Pamd1 are required to prevent the stabilization of early prophase intersister JMs and thereby to favor homolog recombination via intermediates that form in diplonema.

### MCM proteins have diverse functions in meiosis

MCM proteins constitute a conserved eukaryotic protein family of which six members (Mcm2‒7) share three conserved domains (Fig 3). These proteins form a hexameric ring that acts as a DNA helicase to unwind the double helix for the initiation of replication in eukaryotes (see [16]). Some components of the Mcm2‒7 complex may have additional functions unrelated to replication (see [38]). In particular, *Drosophila* Mcm5 was shown to function outside the hexameric complex, since there exists a hypomorphic allele that supports replication initiation but has a distinct meiotic defect by failing to resolve meiotic DSBs into COs [38]. Two members of the wider MCM family, Mcm8 and Mcm9, are not present in all eukaryotes. These proteins are believed to also form heteromultimers that probably act as helicases during replication elongation and recombination-dependent DNA repair ([39] and lit cit. therein; see [17,40]). Mcm8 has additional functions in meiosis that are independent of Mcm9, but possibly require complex formation with other members of the family. Mice without Mcm8 have defective meiotic DSB repair [41]. In *Arabidopsis*, Mcm8 functions in Dmc1-independent, Rad51-dependent DSB repair, probably via the sister chromatid [42]. In *Drosophila*, the Mcm8 homolog, Rec, probably forms a complex with two additional meiosis-specific proteins (Mei-217 and Mei-218) and possibly with some replicative MCM proteins, and functions in the strand invasion step of meiotic recombination [18,39,43]. It was proposed that the Rec complex functionally replaces the Msh4‒Msh5 complex, which in other organisms stabilizes the invading strand against the anti-CO activity of the BLM helicase [18]. In fact, *Drosophila* Mei-217 and Mei-218 are the products of divergent evolution from a single ancestral MCM gene ([18], Fig 3). Mei-218 has a vertebrate homolog, HsMei-218 (a.k.a. Mcmdc2 ‒ Minichromosome Maintenance Domain Containing 2) [18,44,45]. In the mouse, Mcmdc2 is required for the initiation or stabilization of homologous strand invasion; consequently, DSBs remain unrepaired in mutants. Also, Rad51 and Dmc1 foci, which mark early recombination intermediates, persist abnormally in Mcmdc2^‒/‒^ meiocytes [44,45]. Thus, meiotic MCM complexes (possibly of varying composition), while having seemingly different functions in the mouse, in *Arabidopsis*, and in *Drosophila*, may all promote CO or repair by enhancing the processivity of meiotic DNA synthesis in different settings ([39], see [3]).

*Tetrahymena* has clear orthologs of Mcm2–7 replicative helicases. (The *MCM7* gene is currently incorrectly annotated in the Tetrahymena Genome Database (http://ciliate.org/): its actual ID is TTHERM_000011759.) In addition, *Tetrahymena* contains single *MCM9* (TTHERM_00703910) and *MCM8* (TTHERM_01031060) orthologs. Based on their expression profiles (http://tfgd.ihb.ac.cn) these genes seem to have functions in vegetative propagation, although additional meiotic functions for *MCM8* cannot be excluded.

In contrast to the other *Tetrahymena* MCM protein orthologs, Mcmd1 amino acid sequence and secondary structure are strongly diverged and show only limited similarity to meiotic MCM proteins in the other species (Fig 3 and S5 Fig). Similar to the *Drosophila* meiotic MCM complex, *Tetrahymena* Mcmd1 promotes COs but it does so via a different mechanism: Mcmd1, together with Pamd1, retards DNA synthesis and stable D-loop formation to ensure interhomolog CO rather than directly promoting the formation of CO-prone recombination intermediates.

## Materials and methods

### Strains and cell culture

Wild-type *Tetrahymena thermophila* strains B2086 (mating type II) and Cu428 (mating type VII) were obtained from the Tetrahymena Stock Center at Cornell University. Cells were cultured at 30°C using standard methodology [46], and were made competent for mating by starvation in 10 mM Tris HCl (pH7.4) for at least 16 h. Meiosis was induced by mixing starved cultures of wild-type or mutant strains at equal densities (∼2× 10^5^ cells/ml).

### Somatic gene knockout and protein tagging

For somatic gene knockout, (almost) all of the ~50 copies of a target gene in the polyploid somatic macronucleus must be replaced with a deletion cassette carrying an antibiotic resistance marker. Moreover, to investigate the effects of gene inactivation in meiosis, the gene must be deleted in both mating partners because mating cells can share gene products [24]. For the somatic deletion of *MCMD1 and PAMD1*, the respective ORF was replaced with a construct carrying a neomycin or cycloheximide resistance marker under the *MTT1* (metallothionein) promoter [47–49]. Knockout lines were selected in medium with increasing concentrations of cycloheximide or the neomycin derivative paromomycin in the presence of CdCl_2_. (For method details and primer sequences see S2 Text and S3 Table, respectively.) Complete knockout was confirmed by reverse-transcription PCR analysis (S6 Fig).

Mcmd1 and Pamd1 HA-tagged strains were created by fusing a hemagglutinin (HA) coding sequence to the 3´ end of the respective ORF (for details see S2 Text). A strain expressing mCherry-tagged histone H3 was kindly provided by Kensuke Kataoka (Natl Inst. Basic Biol., Okazaki, JP).

### Cell preparation, staining and microscopy

For DAPI (4´,6-diamidino-2-phenylindole) staining, cells were fixed in 4% paraformaldehyde containing 3.4% sucrose and spread onto a slide [15]. For γ-H2A.X immunostaining, cells were dropped onto a slide after fixation with HgCl_2_‒ethanol followed by methanol washes (see [29]). For immunostaining chromatin-associated proteins, cells were treated with Triton X-100 prior to fixation to remove free protein [11], and then primary and fluorescent secondary antibodies were applied. The HA tag, Dmc1 and γ-H2A.X were detected using commercial antibodies (51RAD01 mouse monoclonal, NeoMarkers, Fremont, CA; anti-H2A.X phosphorylated (Ser139) antibody Clone 2F3, BioLegend, San Diego, CA; polyclonal rabbit anti-HA, Sigma-Aldrich, St. Louis, MO).

Fluorescence in situ hybridization (FISH) was performed using a probe produced by amplifying a ~22 kb sequence from the middle of the right arm of micronuclear chromosome 5 and labelling purified PCR products with Cy3 by nick translation. For preparing slides, a pellet from 5 ml cell suspension was resuspended in 1 ml Carnoy’s fixative (methanol‒chloroform‒acetic acid, 6:3:2 ratio). Cells were washed in 70% ethanol, dropped on a slide and air-dried. Chromosomal DNA on slides and the FISH probe and were denatured with hot formamide and hybridized for ~36 h at 37°C (for details see [50]).

Samples on slides were mounted in anti-fading solution (Vector Laboratories, Burlingame, CA) containing DAPI (to stain chromatin). Slides were evaluated by fluorescence microscopy using appropriate filters. Image stacks were recorded using MetaVue software (Molecular Devices, Sunnyvale, CA), deconvolved, false-colored and merged.

Schaudinn fixation plus Giemsa staining [51,52] were used to release nuclei from cells, and the resulting flattened, well-separated chromosomes were inspected by bright-field microscopy.

### Detection of DNA synthesis

A published protocol was used for BrdU incorporation and detection [13]. In short, BrdU was added to a final concentration of 2×10^−4^ M to conjugating cells at 2.5 h after induction of meiosis and cells were harvested at 4 h 15 min after induction of meiosis. Slides of BrdU-fed cells were prepared by the paraformaldehyde‒sucrose method and air-dried. After 3‒5 days, slides were washed with water (5 min) and incubated with 1 M sodium thiocyanate at 90°C for 15 min. The slides were rinsed with 2×SSC and denatured in 70% formamide for 2 min at 68°C to expose the labeled nucleotides to the antibody. Denaturation was stopped by rinsing with ice-cold water, 1×PBS and 1×PBS + 0.05% Triton X-100 for 5 min each. Anti-BrdU antibody (1:40; Abcam, Cambridge, UK) was applied at 4°C overnight, and fluorescence-labeled secondary antibody was applied the next day.

### Protein co-immunoprecipitation

For co-immunoprecipitation (co-IP) experiments, cells were harvested ~3.5 h after meiotic induction (at the stage with maximum nuclear elongation), washed, resuspended in ice-cold Tris lysis buffer (see S2 Text), and ground in a Dounce homogenizer. The cell lysate was filtered and incubated with anti-HA affinity gel for 2 h at 4°C. After washing, proteins eluted from the gel were analyzed by mass-spectrometry (for details see S2 Text).

## Supporting information

S1 Text Production and characterization of *sgs1*(RNAi) *mcmd1*Δ couble mutant cells. (PDF)

S2 Text Supplemental methods. (PDF)

S1 Table Mass spectrometry raw data of Mcmd1 and Pamd1 interacting proteins. (XLSX)

S2 Table BLASTP MCMD1 and PAMD1 homology searches. (PDF)

S3 Table Key resources. (PDF)

S1 Fig Immunoprecipitation of Mcmd1-HA and Pamd1-HA, and co-IP-MS visualization. (A) Western blotting (WB) analysis of Mcmd1-HA and Pamd1-HA immunoprecipitation samples. (B) Visualization of Mcmd1-HA and Pamd1-HA immunoprecipitation proteomics data using scatter plots. The X-axis indicates the enrichment in Mcmd1-HA or Pamd1-HA immunoprecipitation samples compared with control samples. The Y-axis indicates the normalized protein abundance in Mcmd1-HA or Pamd1-HA immunoprecipitation samples using mass spectrometry-based label-free quantification. Raw mass spectrometry data are shown in S1 Table.
(PDF)

S2 Fig BrdU incorporation in *dmc1*Δ meiosis. In the absence of Dmc1, recombination-related (Rad51-dependent) DNA synthesis is not accelerated in fully elongated meiotic prophase nuclei (left mating pairs). DSB repair synthesis takes place only after nuclear shortening (right mating pairs), as in the wild type (Fig 4). The construction of *dmc1*Δ strains was reported in [11].
(PDF)

S3 Fig Localization of γ-H2A.X. Localization of the DSB marker γ-H2A.X (magenta) is not noticeably reduced in elongated meiotic prophase nuclei of the *mcmd1*Δ mutant, indicating that although DSB repair has started (Fig 5), it is not complete at this stage. Five examples of mutant and wild-type mating pairs are shown side by side for direct comparison. Wild-type pairs are distinguished by the expression of tagged histone H3 (cyan) in the MAC of one partner. Mating of a *mcmd1*Δ cell to a wild-type cell rescues the defect in the *mcmd1*Δ cell because Mcmd1 protein can transit through the mating junction. Thus, cells of wild-type (cyan^+^) ‒ mutant (cyan^‒^) pairs are both phenotypically wild type.
(PDF)

S4 Fig BrdU incorporation in *sgs1*RNAi meiotic nuclei. Meiotic DNA synthesis takes place only at diplonema. Red: Anti-BrdU immunostaining. The construction of *sgs1*RNAi strains was reported in [12].
(PDF)

S5 Fig Evolutionary history of MCM family proteins. To construct the maximum-likelihood tree, Mcm7 (representing the conserved MCM replicative helicases), Mcm8, and meiotic MCM domain-containing protein sequences were aligned using the MUSCLE program with default settings [53], and then used to construct the tree in MEGA6 [54]. Branch lengths indicate the number of amino acid substitutions per site.
(PDF)

S6 Fig Reverse-transcription PCR analysis of gene expression. (PDF)
