# Supplementary material for: An MCM family protein promotes interhomolog recombination by preventing precocious intersister repair of meiotic DSBs
Source: PLoS Genet. 2019 Dec 9;15(12):e1008514. doi: 10.1371/journal.pgen.1008514 (PMC6922451; doi:10.1371/journal.pgen.1008514)
Supplement: S1 Text — (PDF) [file pgen.1008514.s001.pdf]

S1 Text. Production and characterization of *sgs1*(RNAi) *mcmd1*Δ double mutant cells

To generate *mcmd1*Δ *sgs1*RNAi double mutant strains, we first generated paromomycin resistant *mcmd1*Δ strains of different mating types by using the co-Deletion method (Hayashi and Mochizuki. 2015. Genetics 201, 55-64). To generate the construct, a 1520 bp DNA fragment from the *MCMD1* 5'-UTR region to the 5' of the ORF was amplified using primers #31-32 and then cloned into the NotI site of the pMcoDel vector by Gibson assembly. For *Tetrahymena* transformation, the circular plasmid DNA was introduced into wild-type mating cells 7 h after induction of mating via biolistic transformation. Transformants were selected by growth in medium containing 0.12 mg/ml paromomycin. After the sexual maturation of transformants, cells with different mating types were isolated for the downstream experiments. A construct for generating *sgs1* RNAi strains (Lukaszewicz et al. 2013. Nucl. Acids Res. 41, 9296-9309) was transformed into starved *mcmd1*Δ cells of different mating types. Transformants were selected by growing in medium containing increasing concentrations (from 15 to 120 µg/ml) of cycloheximide. RNAi was induced by the addition of $CdCl_2$ (Lukaszewicz et al. 2013).

Sgs1 was inducibly depleted in the *mcmd1*Δ background. In pairs of *mcmd1-sgs1* x *mcmd1* cells, one cell undergoes normal anaphase II, whereas the other is mostly unable to undergo the first or second meiotic division. Collapse of anaphase is a characteristic phenotype of *sgs1*Δ (Lukaszewicz et al. 2013). In the double mutant partner, a defect in anaphase I is more frequent, because the separation of univalents (which prevail due to the absence of Mcmd1) during anaphase I is not inhibited. However, the separation of sister chromatids at anaphase II is prevented by the persistence of intersister joint molecules in the absence of Sgs1. (Note that RNAi was not fully effective, since in 30% of mating pairs anphases were not affected in both partners.)

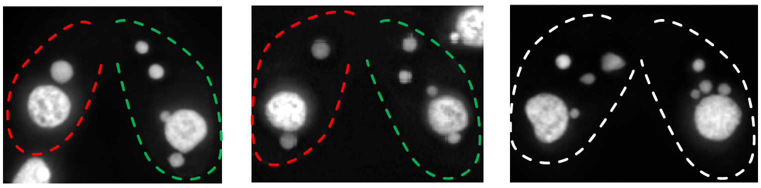

	Anaphase I collapse	Anaphase II collapse		
sgs1(RNAi induced) *mcmd1* x *mcmd1*	20%	50%	30%	n=50
sgs1(RNAi uninduced) *mcmd1* x *mcmd1*	0	0	100%	n=50

Dmc1 was absent from fully elongated nuclei both in the single and the double mutant.

	Dmc1 absent	
sgs1(RNAi induced) *mcmd1* x *mcmd1*	98%	n=100
sgs1(RNAi uninduced) *mcmd1* x *mcmd1*	96%	n=100
