# Supplementary material for: An MCM family protein promotes interhomolog recombination by preventing precocious intersister repair of meiotic DSBs
Source: PLoS Genet. 2019 Dec 9;15(12):e1008514. doi: 10.1371/journal.pgen.1008514 (PMC6922451; doi:10.1371/journal.pgen.1008514)
Supplement: S2 Text — (PDF) [file pgen.1008514.s002.pdf]

S2 Text. Supplemental methods

Somatic gene knockout and protein tagging

To generate the plasmid construct for deleting *MCMD1* from the somatic genome, two ~700 bp fragments flanking the ORF were amplified by PCR (Q5 High-Fidelity DNA Polymerase, New England Biolabs, Beverly, MA) from genomic DNA using the primer pairs #1– #2 and #3– #4, respectively (S3 Table). Using sequence overlaps, the *MCMD1* flanking sequences and the *MTT1* promoter + *CHX* cassette cut out of pChx-SmaI [1] by SmaI digestion were cloned into the NotI site of pBluescript SK(-) by Gibson assembly, using the NEBuilder HiFi DNA Assembly Cloning Kit (New England Biolabs, Beverly, MA). For *Tetrahymena* transformation, the *MCMD1* knockout construct was linearized by NotI digestion and introduced into starved wild-type or mutant cells via biolistic transformation [2]. Transformants were selected by growing in medium containing increasing concentrations (from 15 to 240 µg/ml) of cycloheximide and 4.5 µg/ml $CdCl_2$ (to gradually replace the ~50 wild-type loci in the somatic nucleus with the knockout alleles due to random assortment).

To generate the *PAMD1* somatic knockout construct containing the *MTT1* promoter + *NEO4* cassette, the 5′ and 3′ flanking regions of *PAMD1* were amplified from genomic DNA by PCR using the primer pairs #5– #6 and #7– #8, respectively. The prom*MTT1-NEO4* cassette containing adapter sequences for overlapping PCR was amplified from the pNeo4 plasmid [3] by PCR using the primer pair #9– #10. The *PAMD1* flanking sequences and the prom*MTT1-NEO4* cassette were then fused together by overlapping PCR using the primer pair #5– #8. The vector backbone DNA was amplified from the pJET1.2 blunt vector (Thermo Fisher Scientific, Waltham, MA) by PCR using the primer pair #11– #12. Finally, the prom*MTT1-NEO4* cassette containing *PAMD1*-flanking sequences was cloned into the pJET1.2 backbone by Gibson assembly. The knockout construct was linearized by XhoI–XbaI double digestion and introduced into starved wild-type or mutant cells via biolistic transformation. Transformants were selected in medium containing decreasing concentrations of $CdCl_2$ (from 1 to 0.1 µg/ml) and increasing concentrations of paromomycin (0.12–16 mg/ml) until the wild-type chromosomes were completely replaced by the knockout chromosomes in the somatic nucleus [2].

Somatic gene knockout candidates were first screened with quantitative PCR using genomic DNA as the template (data not shown). The lack of mRNA expression from the target locus was confirmed by RT-PCR. In brief, total RNA of mating knockout cells and mating wild-type cells was extracted 4 h after induction of meiosis, using the peqGOLD TriFast solution (PEQLAB, Erlangen, Germany). Next, 1 µg of total RNA was treated with ezDNase enzyme (Thermo Fisher Scientific, Waltham, MA) to remove genomic DNA and used for cDNA synthesis using the SuperScript IV VILO Master Mix (Thermo Fisher Scientific, Waltham, MA), according to the instructions of the manufacturer. Finally, by using cDNA as the template, mRNA expression from *MCMD1*, *PAMD2*, and *HOP2* loci was inspected by PCR with

primer pairs that bind to adjacent exons (S3 Table). mRNA expression from the *TWI1* locus, which served as a control for loading and proper cell cycle stage, was also inspected in each sample (S6 Fig).

Plasmid constructs for expressing C-terminally HA-tagged Mcmd1 or Pamd1 from the endogenous promoter were generated as follows: For *MCMD1*, DNA fragments were amplified from the ORF and the 3′ flanking region using primer pairs #13–#14 and #15–#16, respectively. The fragments were fused to a prom*MTT1*-EGFP-tag sequence-containing DNA fragment that had been excised from the pEGFP-*NEO4* plasmid [4] using BamHI–XhoI double digestion, and then cloned into the SacI–KpnI sites of pBluescript SK(+) using Gibson assembly. Next, the EGFP-tag sequence was cut out of this plasmid by BamHI–PstI double digestion and replaced with the HA sequence excised from the pHA-*neo4* plasmid using the same set of restriction enzymes. To replace the *NEO4* resistance marker with a *CHX* cassette, the *NEO4* cassette was cut out using PstI–SalI double digestion. The remainder of the linearized DNA fragment was fused to a *CHX* cassette (amplified from the p*CHX*-SmaI plasmid by PCR using the primer pair #17– #18) by Gibson assembly. The Pamd1-HA tagging construct was generated in the same way, but without replacement with the *CHX* cassette. For transformation, these constructs were linearized by SacI–KpnI double digestion and introduced into starved wild-type cells or knockout mutants via biolistic transformation as described above.

Co-immunoprecipitation and mass spectrometry data analysis

For co-immunoprecipitation, 400 ml of conjugating cells ($\sim 5\times10^5$ cells/ml) expressing C-terminally HA-tagged Mcmd1 or Pamd1 protein were harvested 3.5 h after mixing, resuspended in 10 ml ice-cold lysis buffer (30 mM Tris-Base, 20 mM KCl, 2 mM $MgCl_2$, 150 mM NaCl, 0.1% Triton X-100, 1 mM phenylmethylsulfonylfluoride (PMSF), and cOmplete EDTA-free protease inhibitor (Roche, Indianapolis, IN), pH8.1), and lysed with a 15 ml Dounce tissue grinder (Kimber Chase, Vineland, NJ). The cell lysate was filtered and incubated with 100 µl EZview Red Anti-HA Affinity Gel (Sigma-Aldrich, St Louis, MO) for 2 h at 4°C. After washing four times with 1 ml wash buffer (30 mM Tris-Base, 20 mM KCl, 2 mM $MgCl_2$, 1 mM PMSF, cOmplete protease inhibitor, pH8.1; three times in the presence of 0.1% Triton X-100 and once without), proteins associated with the anti-HA antibody were eluted by incubating the gel beads in 400 µl wash buffer containing 0.1% Triton X-100 and 250 µg/ml HA peptide at room temperature for 20 min. Eluates were precipitated with 10% trichloroacetic acid (TCA) and boiled in 1× SDS-loading buffer (50 mM Tris-HCl pH6.8, 2% sodium dodecyl sulfate, 10% glycerol, 1% β-mercaptoethanol, 0.02% bromophenol blue). A small aliquot was loaded onto a SDS-PAGE gel for western blotting with a mouse anti-HA monoclonal antibody (1:1000 dilution; clone HA-7; Sigma-Aldrich) to confirm precipitation (S1 Fig A).

To identify co-immunoprecipitated proteins, TCA-precipitated samples were run into an SDS-PAGE gel for 2 cm and separated protein bands were stained with Coomassie-Blue and excised for tryptic

digestion. After digestion, the peptides were separated on an Ultimate 3000 RSLC nano-flow chromatography system and analyzed on a Q Exactive HF Orbitrap mass spectrometer, equipped with a Proxeon nanospray source (all from Thermo Fisher Scientific). Raw data were processed using MaxQuant software [5] and analyzed with the Perseus software package [6]. Instrument settings for mass spectrometry analysis and raw proteomics data were deposited at the PRIDE [7] repository of the ProteomeXchange Consortium with the dataset identifier PXD015986.

To identify proteins enriched in the co-immunoprecipitation samples, the label-free quantification method was used for protein quantification [8]. Proteins with a Log2 ratio for sample/control intensity of >3 and a peptide spectra count of >5 were considered to be enriched proteins (S1 Fig and S1 Table).

Reference List

1. Gao S, Xiong J, Zhang C, Berquist BR, Yang R, Zhao M, Molascon AJ, Kwiatkowski SY, Yuan D, Qin Z, Wen J, Kapler GM, Andrews PC, Miao W, Liu Y (2013) Impaired replication elongation in *Tetrahymena* mutants deficient in histone H3 lysine 27 mono-methylation. Genes Dev 27: 1662-1679.

2. Cassidy-Hanley D, Bowen J, Lee JH, Cole E, VerPlank LA, Gaertig J, Gorovsky MA, Bruns PJ (1997) Germline and somatic transformation of mating *Tetrahymena thermophila* by particle bombardment. Genetics 146: 135-147.

3. Mochizuki K (2008) High efficiency transformation of *Tetrahymena* using a codon-optimized neomycin resistance gene. Gene 425: 79-83.

4. Kataoka K, Schoeberl UE, Mochizuki K (2010) Modules for C-terminal epitope tagging of *Tetrahymena* genes. J Microbiol Meth 82: 342-346.

5. Cox J, Mann M (2008) MaxQuant enables high peptide identification rates, individualized p.p.b.-range mass accuracies and proteome-wide protein quantification. Nature Biotechnol 26: 1367-1372.

6. Tyanova S, Temu T, Sinitcyn P, Carlson A, Hein MY, Geiger T, Mann M, Cox J (2016) The Perseus computational platform for comprehensive analysis of (prote)omics data. Nature Meth 13: 731-740.

7. Perez-Riverol Y, Csordas A, Bai J, Bernal-Llinares M, Hewapathirana S, Kundu DJ, Inuganti A, Griss J, Mayer G, Eisenacher M, Pérez E, Uszkoreit J, Pfeuffer J, Sachsenberg T, Yilmaz S, Tiwary S, Cox J, Audain E, Walzer M, Jarnuczak AF, Ternent T, Brazma A, Vizcaíno JA (2019) The PRIDE database and related tools and resources in 2019:improving support for quantification data. Nucl Acids Res 47: D422-D450.

8. Cox J, Hein MY, Luber CA, Paron I, Nagaraj N, Mann M (2014) Accurate proteome-wide label-free quantification by delayed normalization and maximal peptide ratio extraction, termed MaxLFQ. Mol Cell Proteomics 13: 2513-2526.
