# Supplementary material for: An MCM family protein promotes interhomolog recombination by preventing precocious intersister repair of meiotic DSBs
Source: PLoS Genet. 2019 Dec 9;15(12):e1008514. doi: 10.1371/journal.pgen.1008514 (PMC6922451; doi:10.1371/journal.pgen.1008514)
Supplement: S2 Table — (PDF) [file pgen.1008514.s004.pdf]

**S2 Table. Mcmd1 and Pamd1 BLASTP searches**

| Query Protein | Group | | Species | Top hit (ID) | BLASTP E-value* |
|---|---|---|---|---|---|
| | | | *Homo sapiens* | Mcm8 (sp\|Q9UJA3-4\|MCM8_HUMAN) | 0.024 |
| | | | *Mus musculus* | Mcm8 (sp\|Q9CWV1\|MCM8_MOUSE) | 0.001 |
| | Multicelluar animals | | *Danio rerio* | Mcm4 (ENSDARP00000097832) | 2.64E-04 |
| | | | *Drosophila melanogaster* | Rec (FBpp0306662) | 1.98E-05 |
| | | | *Caenorhabditis elegans* | Mcm4 (Y39G10AR.14a) | 3.26E-06 |
| | Land plants | | *Aradidopsis thaliana* | Mcm8 (AT3G09660.1) | 0.61 |
| | Fungi | | *Saccharomyces cerevisiae* | Mcm4 (YPR019W) | 2.22E-04 |
| | | | *Schizosaccharomyces pombe* | Sre2 (SPBC354.05c.1:pep) | 1.7 |
| | | | *Tetrahymena borealis* | EI9_20942.1 | 0 |
| **Mcmd1** | | *Tetrahymena* | *Tetrahymena elliotti* | EI7_15054.1 | 0 |
| | | | *Tetrahymena malaccensis* | EIA_23651.1 | 0 |
| | | | *Paramecium tetraurelia* | Mini-chromosome maintenance, DNA-dependent ATPase (GSPATP00012268001) | 1.2 |
| | | Other ciliates | *Ichthyophthirius multifilis* | mcm2-3-5 family protein, putative (IMG5_106700) | 0.41 |
| | Protists | | *Oxytricha trifallax* | MCM8a (Contig7673.0.g5) | 0.075 |
| | | | *Stylonychia lemnae* | mcm family protein (Contig915.g1014) | 0.054 |
| | | | *Plasmodium falciparum* | Mcm5 (PF3D7_1211700) | 2.9 |
| | | Other protists | *Toxoplasma gondii* | Mcm5 (TGME49_243920) | 0.003 |
| | | | *Trypanosoma brucei* | MCM complex subunit, putative (Tbg972.11.13060) | 0.14 |
| | | | *Giardia lamblia* | Mcm5 (GL50581_282) | 0.17 |
| | | | *Homo sapiens* | Cilia And Flagella Associated Protein 53 (sp\|Q96M91\|CFA53_HUMAN) | 0.064 |
| | | | *Mus musculus* | N/A | N/A |
| | Multicelluar animals | | *Danio rerio* | Radial spoke head 3 homolog (ENSDARP00000017411) | 1 |
| | | | *Drosophila melanogaster* | Mei218 (FBpp0292855) | 0.16 |
| | | | *Caenorhabditis elegans* | T23F2.2b | 4.8 |
| | Land plants | | *Aradidopsis thaliana* | N/A | N/A |
| | Fungi | | *Saccharomyces cerevisiae* | YAP5 (YIR018W) | 1.4 |
| | | | *Schizosaccharomyces pombe* | Cell polarity protein alp21 (SPAC22H10.10.1:pep) | 1.7 |
| | | | *Tetrahymena borealis* | EI9_18594.1 | 0 |
| **Pamd1** | | *Tetrahymena* | *Tetrahymena elliotti* | EI7_16247.1 | 0 |
| | | | *Tetrahymena malaccensis* | EIA_22197.1 | 0 |
| | | | *Paramecium tetraurelia* | Protein kinase-like domain (GSPATP00021108001) | 5.6 |
| | | Other ciliates | *Ichthyophthirius multifilis* | Hypothetical protein (IMG5_120920) | 1.1 |
| | Protists | | *Oxytricha trifallax* | GCC2 and GCC3 (Contig473.1.g50) | 0.057 |
| | | | *Stylonychia lemnae* | Eukaryotic translation initiation factor 4e type 2 (Contig160.g191) | 0.64 |
| | | | *Plasmodium falciparum* | DEAD box ATP-dependent RNA helicase, putative (PF3D7_0411400) | 0.86 |
| | | Other protists | *Toxoplasma gondii* | Phospholipase, patatin family protein (TGME49_231370) | 8.3 |
| | | | *Trypanosoma brucei* | Hypothetical protein, conserved (Tbg972.8.460) | 3.2 |
| | | | *Giardia lamblia* | Hypothetical protein (GL50581_1233) | 0.95 |

*BLASTP E-value threshold was set to 10 (default). Homologs with an E-value equal to or lower than 0.001 (1e-3) are colored in blue.
