# Supplementary material for: An MCM family protein promotes interhomolog recombination by preventing precocious intersister repair of meiotic DSBs
Source: PLoS Genet. 2019 Dec 9;15(12):e1008514. doi: 10.1371/journal.pgen.1008514 (PMC6922451; doi:10.1371/journal.pgen.1008514)
Supplement: S3 Table — (PDF) [file pgen.1008514.s005.pdf]

S3 Table. Key resources

Primer ID	Primer full name	Purpose	Primer sequence (5' -> 3', adapter sequence used for ligation)
#1	Mcmd1_5UTRf162_NotI	*MCMD1* somatic gene knockout	AGTTCTAGAGCGGCCGC ATCTTGCTTTATCAATCATTCATTG
#2	Mcmd1_5UTRr1061_N4		GTCAGGTGCCTGGTACCC TTGCTAACTTAGACAGAAATTCAG
#3	Mcmd1_3UTRf3866_N4		CTGACGTCGCACCATCCC GTGAAATAAAACTTCTATTACCAAG
#4	Mcmd1_3UTRr4591_Not1		ACCGCGGTGGCGGCCGC TCTAAGTCAAGTTCTTTCCTTAC
#5	891_5´fl FW	*PAMD1* somatic gene knockout	ctcgagtttttcagcaagat GGCTTGCTTGAATGAATG
#6	891_5´fl RV		cctgcagccc CTAATTTGAATCGACTTCTATAAGTTAC
#7	891_3´fl FW		aatgcagccc CTCCATCTCCTTTCCTAAAAAATTTC
#8	891_3´fl RV		aggagatcttctagaaagat GTGCTTTTATAGATCAATATTGTTG
#9	891_NEO4_Kaz_Fwd		ttcaaattag GGGCTGCAGGAATTCGATA
#10	891_NEO4_Kaz_ Rev		ggagatggag GGGCTGCATTTTTCCAGTAAAAATTTG
#11	pJet_Fwd		ATCTTTCTAGAAGATCTCCTAC
#12	pJet_Rev		ATCTTGCTGAAAAACTCGAG
#13	Mcmd1_cdsf3099_SacG	Mcmd1 C-terminal HA tagging	ACTAAAGGGAACAAAAGCTGGAGCTC ATTTCTTAAATAAGTTCCAACT[illegible]
#14	Mcmd1_cdsr3840_BamH1G		TCTTCACCCTTAGAAACCATGGATCC TAGCTTATCTAAGTTAAGCTAA[illegible]
#15	Mcmd1_3UTRf3866_Xho1G		GCTTATCGATACCGTCGACCTCGAG GTGAAATAAAACTTCTATTACCA[illegible]
#16	Mcmd1_3UTRr4591_Kpn1Gib		CTCACTATAGGGCGAATTGGGTACC TCTAAGTCAAGTTCTTTCCTTAC
#17	Chx_fw		TCCAGATCCCCCGGGCTGCAG taccaggcacctgacgg
#18	Chx_rv		AGTTTTATTTCACCTCGAGGTCGAC atggtgcgacgtcagtttttcc
#19	Mcdp_CDSf2863_SacG	Pamd1 C-terminal HA tagging	ACTAAAGGGAACAAAAGCTGGAGCTC TCAGTATTGTCTTTAAAGGATT[illegible]
#20	Mcdp_CDSr3578_BamG		TCTTCACCCTTAGAAACCATGGATCC AAATCTTATAAAATCTAAAATC[illegible]
#21	Mcdp_3UTRf3607_XhoG		GCTTATCGATACCGTCGACCTCGAG TAAGTTACTCAAAGATAAAACAG[illegible]
#22	Mcdp_3UTRr4321_KpnG		CTCACTATAGGGCGAATTGGGTACC TGCTTGAATGAATGAAGTAGG
#23	Mcmd1_KOckf3197	*MCMD1* knockout validation	ATCTGAATTAGTGCGTTAAGC
#24	Mcmd1_KOckr3628		TAATTTTTCTACCTGTTACTTCTTC
#25	Pamd1_KOckf3363	*PAMD1* knockout validation	ATTTTGTAGAGATTTAGACACAAAG
#26	Pamd1_KOckr3563		TAAAATCTAATCTTAAATCTTCTCC
#27	Hop2_Cdsqf2194	*HOP2* knockout validation	ATGAAAATACTGATGAAAGTGATAG
#28	Hop2_Cdsqr2553		TTTAAGTTACTTTCAAGAGTAGTTG
#29	Twi1_CDSf3018	*TWI1* RT-PCR	TTTAGCAAGTTTATGAATAAGCTC
#30	Twi1_CDSr3574		TAATAGTGTAGTGAGTAGGAGAAGC
#31	7610 5´ coDel	*MCMD1* somatic gene knockout by using co-Deletion	CTTTATTGTTATCATCTTATGACCGC gggttagctagaagagttc
#32	7610 3´ coDel		CTCATCAAGTTGTAATGCTAAAATGCctgtctaagttagcaagc

Plasmids	Note	Source
pChx_SmaI		Gao et al. Genes&Dev, 2013
pBluescript SK (-)	GenBank: X52324.1	Stratagene
pMcoDel		Noto et al. Mol Cell, 2015
pSGS1 RNAi		Lukaszewicz et al. Nucleic Acids Res, 2013
pNeo4	GenBank: EU606202.1	Mochizuki. Gene, 2008
pBluescript SK (+)	GenBank: X52325.1	Stratagene
pHA-neo4	GenBank:AB570108	Kataoka et al. Gene, 2010
pEGFP-neo4	GenBank:AB570109	Kataoka et al. Gene, 2010
pJET1.2 blunt		Thermo Fisher (Fermentas)

T. thermophila **strains**	Note	Source
B2086 II	Wild-type; Mating type II	Tetrahymena Stock Center; Strain ID: SD01625
CU428.2	Wild-type; Mating type IIV	Tetrahymena Stock Center; Strain ID: SD00178
*hop2*Δ B2086		Mochizuki. Gene, 2008
[illegible]		
