# Supplementary material for: An MCM family protein promotes interhomolog recombination by preventing precocious intersister repair of meiotic DSBs
Source: PLoS Genet. 2019 Dec 9;15(12):e1008514. doi: 10.1371/journal.pgen.1008514 (PMC6922451; doi:10.1371/journal.pgen.1008514)
Supplement: S1 Fig — (A) Western blotting (WB) analysis of Mcmd1-HA and Pamd1-HA immunoprecipitation samples. (B) Visualization of Mcmd1-HA and Pamd1-HA immunoprecipitation proteomics data using scatter plots. The X-axis indicates the enrichment in Mcmd1-HA or Pamd1-HA immunoprecipitation samples compared with control samples. The Y-axis indicates the normalized protein abundance in Mcmd1-HA or Pamd1-HA immunoprecipitation samples using mass spectrometry-based label-free quantification. Raw mass spectrometry data are shown in S1 Table. (PDF) [file pgen.1008514.s006.pdf]

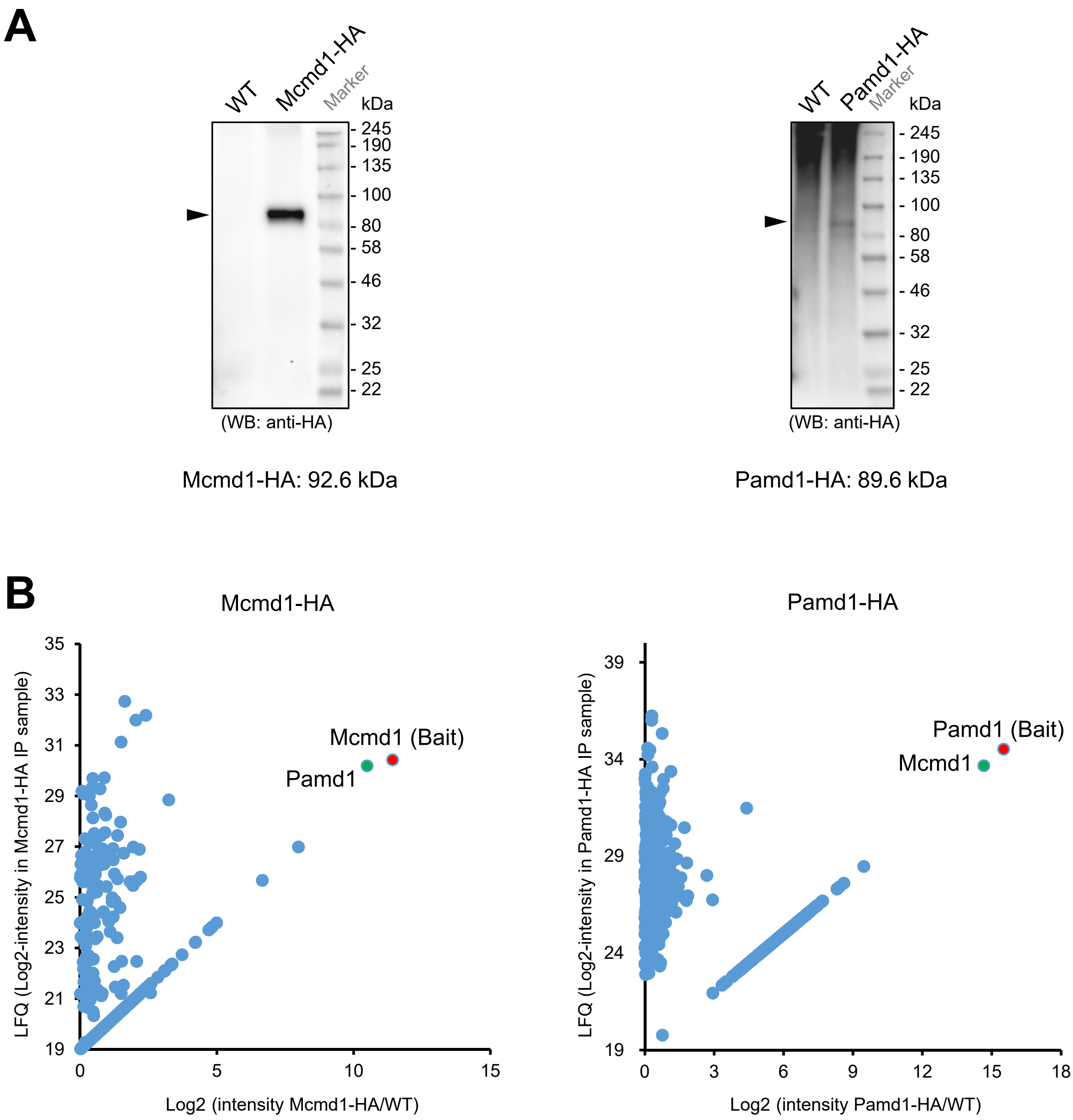

S1 Fig. Immunoprecipitation of Mcmd1-HA and Pamd1-HA, and co-IP-MS visualization. (A) Western blotting (WB) analysis of Mcmd1-HA and Pamd1-HA immunoprecipitation samples. **(B)** Visualization of Mcmd1-HA and Pamd1-HA immunoprecipitation proteomics data using scatter plots. The X-axis indicates the enrichment in Mcmd1-HA or Pamd1-HA immunoprecipitation samples compared with control samples. The Y-axis indicates the normalized protein abundance in Mcmd1-HA or Pamd1-HA immunoprecipitation samples using mass spectrometry-based label-free quantification. Raw mass spectrometry data are shown in S1 Table.
