# Supplementary figures and images for: An MCM family protein promotes interhomolog recombination by preventing precocious intersister repair of meiotic DSBs
Source: PLoS Genet. 2019 Dec 9;15(12):e1008514. doi: 10.1371/journal.pgen.1008514 (PMC6922451; doi:10.1371/journal.pgen.1008514)

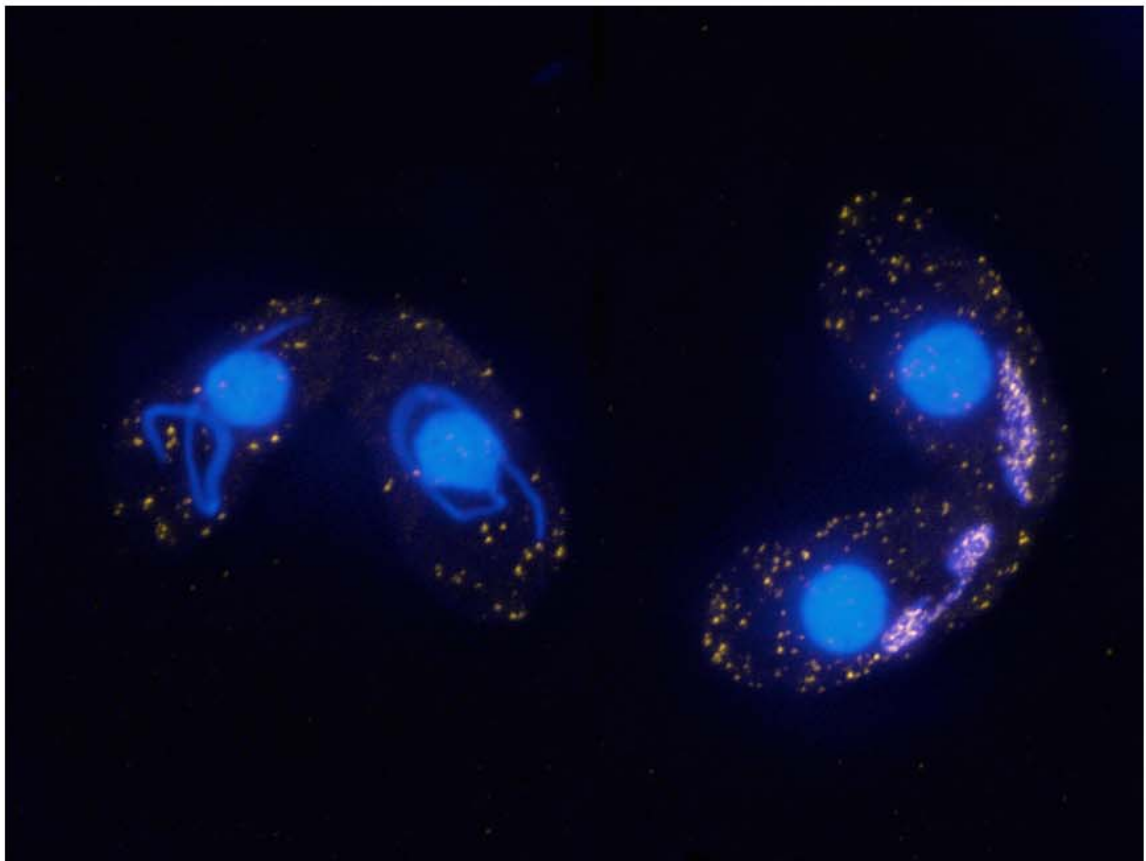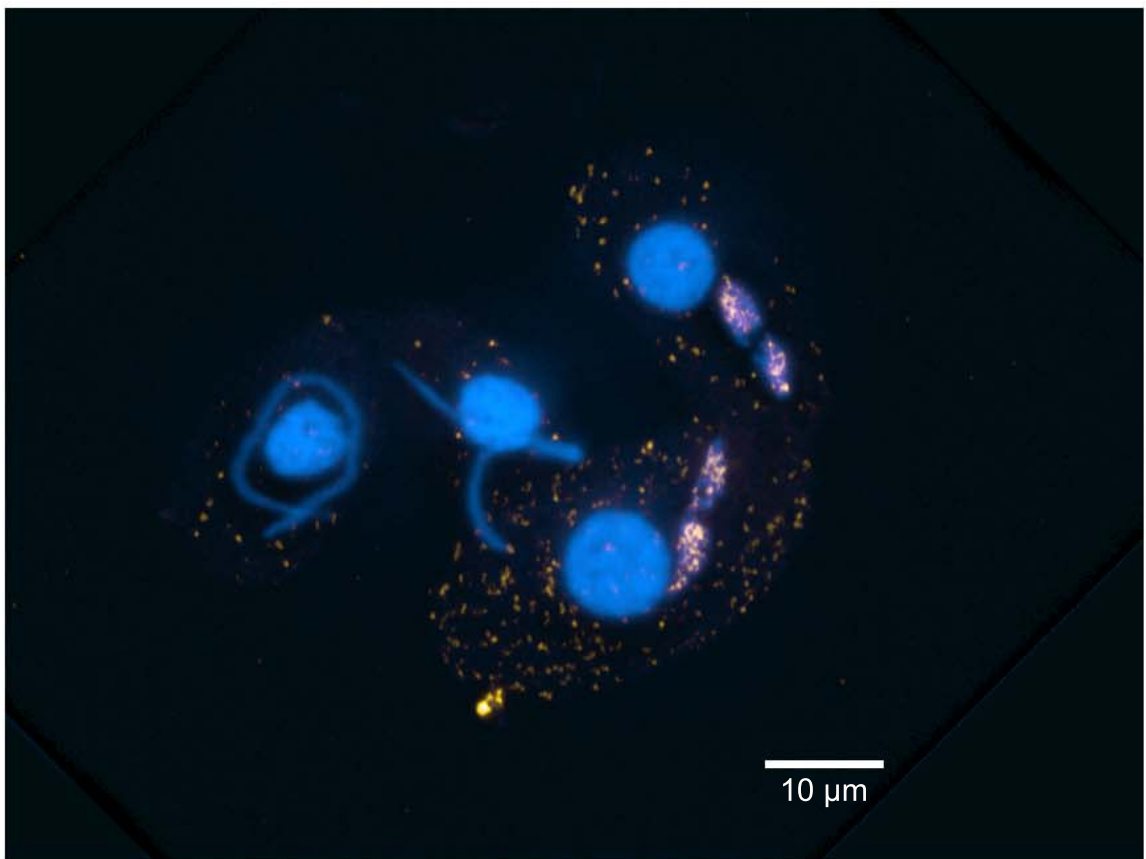

S2 Fig

Supplement: S2 Fig — In the absence of Dmc1, recombination-related (Rad51-dependent) DNA synthesis is not accelerated in fully elongated meiotic prophase nuclei (left mating pairs). DSB repair synthesis takes place only after nuclear shortening (right mating pairs), as in the wild type (Fig 4). The construction of dmc1Δ strains was reported in [11]. (PDF) [file pgen.1008514.s007.pdf]

S3 Fig

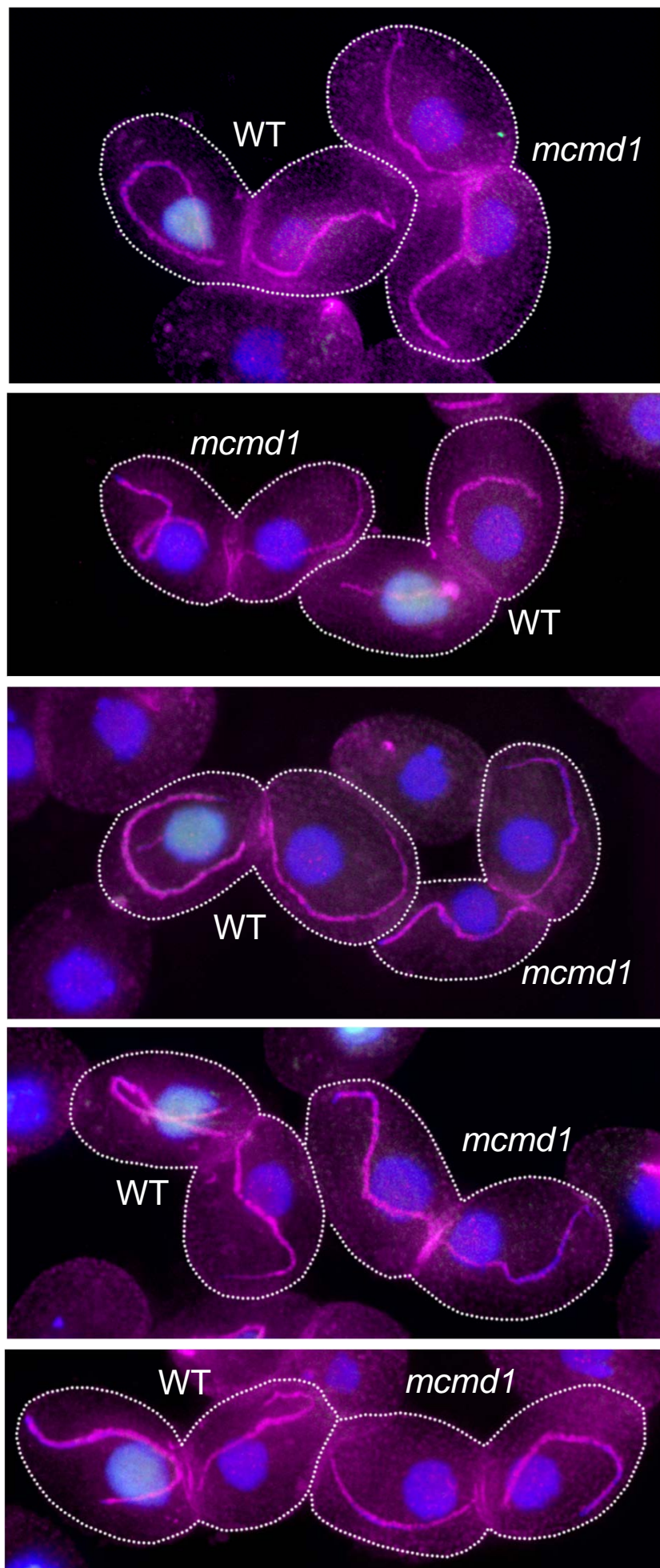

Supplement: S3 Fig — Localization of the DSB marker γ-H2A.X (magenta) is not noticeably reduced in elongated meiotic prophase nuclei of the mcmd1Δ mutant, indicating that although DSB repair has started (Fig 5), it is not complete at this stage. Five examples of mutant and wild-type mating pairs are shown side by side for direct comparison. Wild-type pairs are distinguished by the expression of tagged histone H3 (cyan) in the MAC of one partner. Mating of a mcmd1Δ cell to a wild-type cell rescues the defect in the mcmd1Δ cell because Mcmd1 protein can transit through the mating junction. Thus, cells of wild-type (cyan+) ‒ mutant (cyan‒) pairs are both phenotypically wild type. (PDF) [file pgen.1008514.s008.pdf]

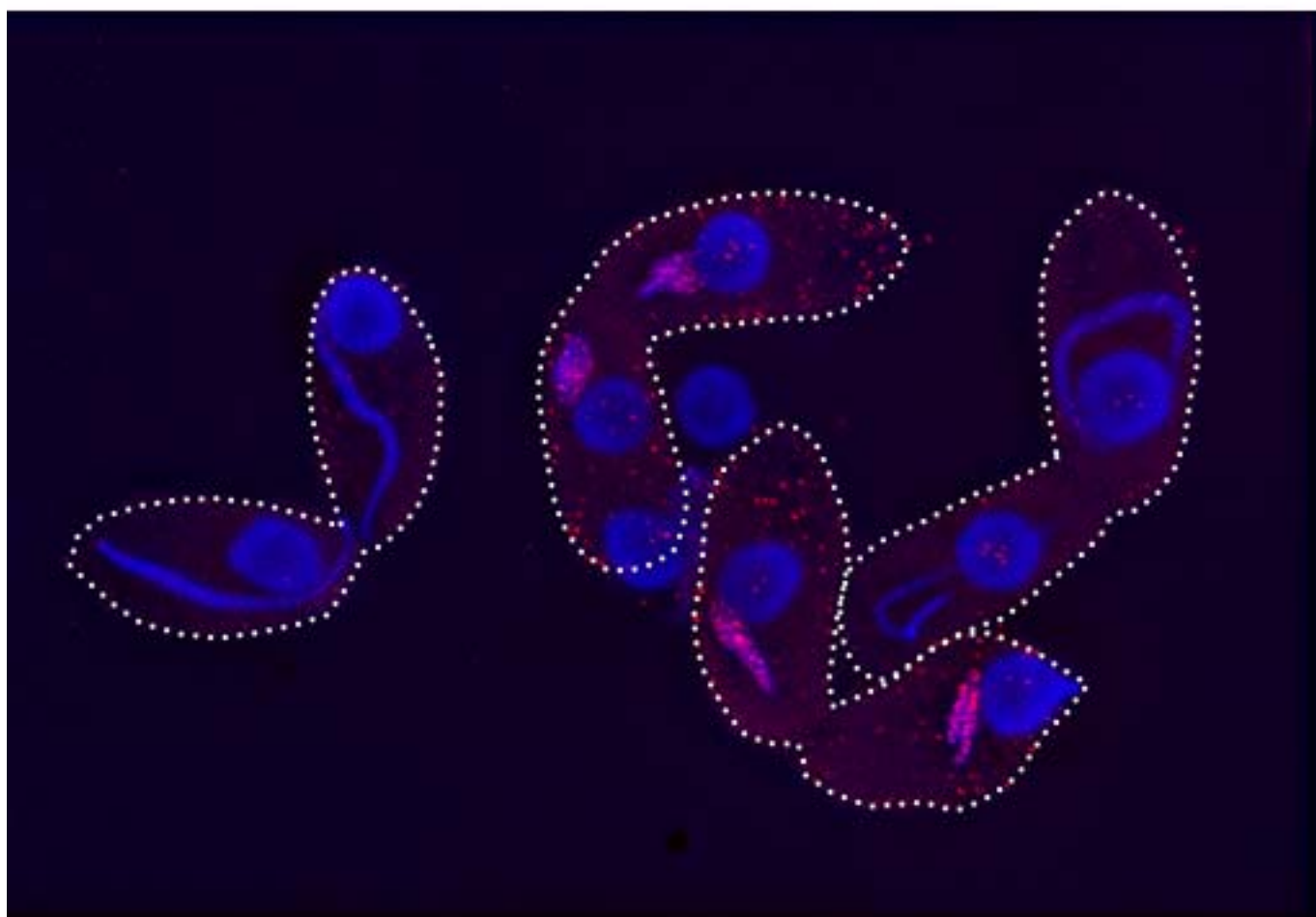

S4 Fig

Supplement: S4 Fig — Meiotic DNA synthesis takes place only at diplonema. Red: Anti-BrdU immunostaining. The construction of sgs1RNAi strains was reported in [12]. (PDF) [file pgen.1008514.s009.pdf]

**S5 Fig.** Evolutionary history of MCM family proteins

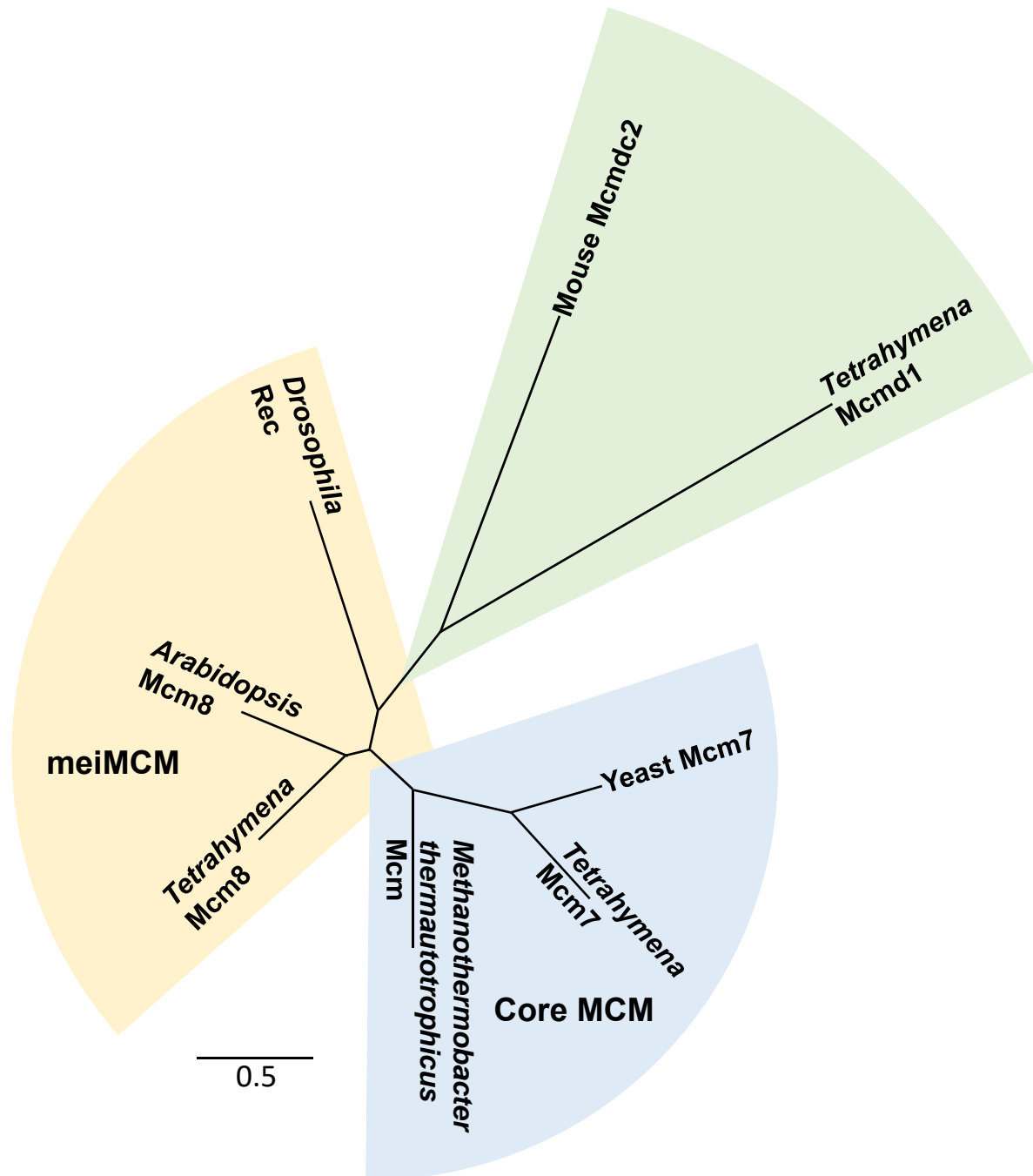

Supplement: S5 Fig — To construct the maximum-likelihood tree, Mcm7 (representing the conserved MCM replicative helicases), Mcm8, and meiotic MCM domain-containing protein sequences were aligned using the MUSCLE program with default settings [53], and then used to construct the tree in MEGA6 [54]. Branch lengths indicate the number of amino acid substitutions per site. (PDF) [file pgen.1008514.s010.pdf]
