# Supplementary material for: An MCM family protein promotes interhomolog recombination by preventing precocious intersister repair of meiotic DSBs
Source: PLoS Genet. 2019 Dec 9;15(12):e1008514. doi: 10.1371/journal.pgen.1008514 (PMC6922451; doi:10.1371/journal.pgen.1008514)
Supplement: S6 Fig — (PDF) [file pgen.1008514.s011.pdf]

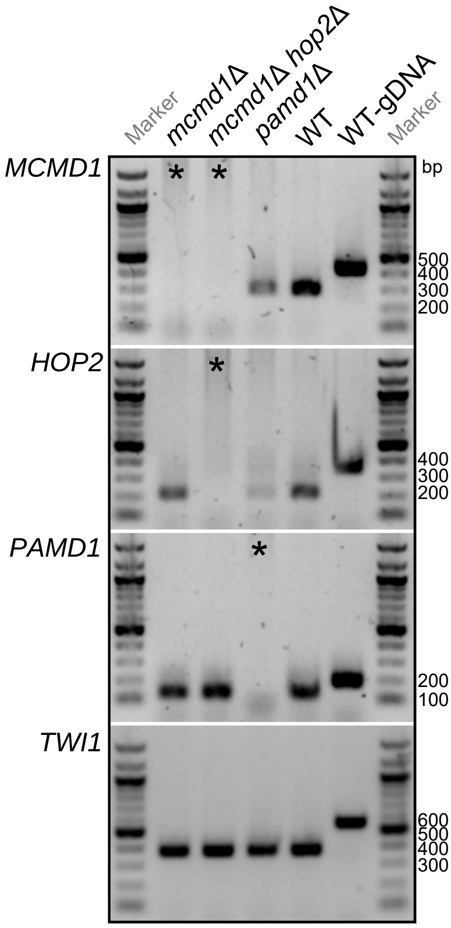

Gene	PCR product size (bp) gDNA	cDNA
MCMD1	432	299
PAMD1	201	142
HOP2	360	214
TWI1	557	372

S6 Fig. Reverse-transcription PCR analysis of gene expression in wild-type, *mcmd1Δ*, *mcmd1Δ hop2Δ,* and *pamd1Δ* cells in meiotic prophase.

Left panel: Expression of *MCMD1*, *HOP2*, *PAMD1*, and *TWI1* was investigated by RT-PCR, using cDNA samples generated from total RNAs of wild-type (WT), *mcmd1Δ, mcmd1Δ hop2Δ,* and *pamd1Δ* cells at four hours after induction of meiosis. To ensure that PCR products were amplified from cDNAs, primer pairs that bind to adjacent exons were used for PCR. PCR products amplified from *Tetrahymena* genomic DNA (WT-gDNA) were used as controls for the size of intron-containing DNA fragments. The asterisk indicates absence of gene expression. Right panel: PCR product size information. *TWI1* served as a control for the correct staging of samples. PCR primer sequences are listed in S3 Table.
